# Supplementary material for: Critical assessment of LC3/GABARAP ligands used for degrader development and ligandability of LC3/GABARAP binding pockets
Source: Nat Commun. 2024 Nov 25;15:10204. doi: 10.1038/s41467-024-54409-5 (PMC11589570; doi:10.1038/s41467-024-54409-5)
Supplement: Supplementary file 1 — Supplementary Information [file 41467_2024_54409_MOESM1_ESM.pdf]

Supplementary information

Critical assessment of LC3/GABARAP ligands used for degrader development and ligandability of LC3/GABARAP binding pockets

Martin P. Schwalm[1,2,3], Johannes Dopfer[1,2], Adarsh Kumar[1], Francesco A. Greco[1,2], Nicolas Bauer[1,2], Frank Löhr[4], Jan Heering[5], Sara Cano-Franco[6,7], Severin Lechner[8], Thomas Hanke[1,2], Ivana Jaser[9], Viktoria Morasch[1,2], Christopher Lenz[1,2], Daren Fearon[10], Peter G. Marples[10], Charles W. E. Tomlinson[10], Lorene Brunello[6,7], Krishna Saxena[1,2], Nathan B. P. Adams[9], Frank von-Delft[10], Susanne Müller[1,2], Alexandra Stolz[6,7], Ewgenij Proschak[1,5], Bernhard Kuster[8], Stefan Knapp[1,2,3*], Vladimir V. Rogov[1,2*]

[1]Institute for Pharmaceutical Chemistry, Department of Biochemistry, Chemistry and Pharmacy, Goethe University Frankfurt, Max-von-Laue-Straße 9, 60438 Frankfurt, Germany

[2]Structural Genomics Consortium, Buchmann Institute for Molecular Life Sciences, Goethe University Frankfurt, Max-von-Laue-Straße 15, 60438 Frankfurt, Germany

[3]German Cancer Consortium (DKTK) / German Cancer Research Center (DKFZ), DKTK site Frankfurt-Mainz, 69120 Heidelberg, Germany

[4]Institute for Biophysical Chemistry, Department of Biochemistry, Chemistry and Pharmacy, Goethe University Frankfurt, Max-von-Laue-Straße 9, 60438 Frankfurt, Germany

[5]Fraunhofer Institute for Translational Medicine and Pharmacology ITMP, Theodor-Stern-Kai 7, 60596 Frankfurt, Germany

[6]Institute of Biochemistry II (IBC2), Faculty of Medicine, Goethe University, Theodor-Stern-Kai 7, 60590, Frankfurt am Main, Germany.

[7]Buchmann Institute for Molecular Life Sciences (BMLS), Goethe University, Max-von-Laue-Straße 15, 60438, Frankfurt am Main, Germany.

[8]Chair of Proteomics and Bioanalytics, TUM School of Life Sciences, Technical University of Munich, 85354 Freising, Germany

[9]NanoTemper Technologies GmbH, Flößergasse 4, 81369 Munich, Germany.

[10]Diamond Light Source Ltd., Harwell Science and Innovation Campus, Didcot OX11 0QX, UK.

*correspondence: knapp@pharmchem.uni-frankfurt.de, rogov@pharmchem.uni-frankfurt.de

Table of content:

Supplementary Figures S4-S23

Supplementary Figure 1. Chemical structures of the compounds used in this study. S4

Supplementary Figure 2. Establishment of diverse binding assays for all human Atg8 homologs (LC3/GABARAPs). S5

Supplementary Figure 3. Interaction between LC3B and compounds **12** and **1-4** investigated by NMR. S7

Supplementary Figure 4. Characterization of compounds binding to LC3/GABARAP proteins. S9

Supplementary Figure 5. Interaction between GABARAPL2 and compounds **1-4** investigated by NMR. S11

Supplementary Figure 6. ESI mass spectrometry for all LC3/GABARAP proteins treated with compounds **5-7.** S13

Supplementary Figure 7. ESI mass spectrometry for GABARAPL2 treated with different concentrations of compound **4.** S15

Supplementary Figure 8. Cellular assays. S16

Supplementary Figure 9. Microscopy images. S18

Supplementary Figure 10. Interaction between LC3B/GABARAP proteins and compound **21.** S19

Supplementary Figure 11. Results of TH152 (**21**) docking onto the LC3B structure with interacting residues marked as determined by NMR**.** S21

Supplementary Figure 12. Chemical properties of virtually screened compounds and distribution of resulting scores. S22

Supplementary Figure 13. Electron densities of selected compounds in the corresponding LC3B structures. S24

Supplementary Tables S25-S32

Supplementary Table 1. Structure-activity relationship of sulfonamide-based compounds. S25

Supplementary Table 2. Structure-activity relationship of benzothiazole based compounds. S26

Supplementary Table 3. Structure-activity relationship of pyrimidine-based compounds. S27

Supplementary Table 4. X-ray data collection and refinement statistics. S29

Supplementary Table 5. Small molecule screening data. S33

Supplementary References S34-S35

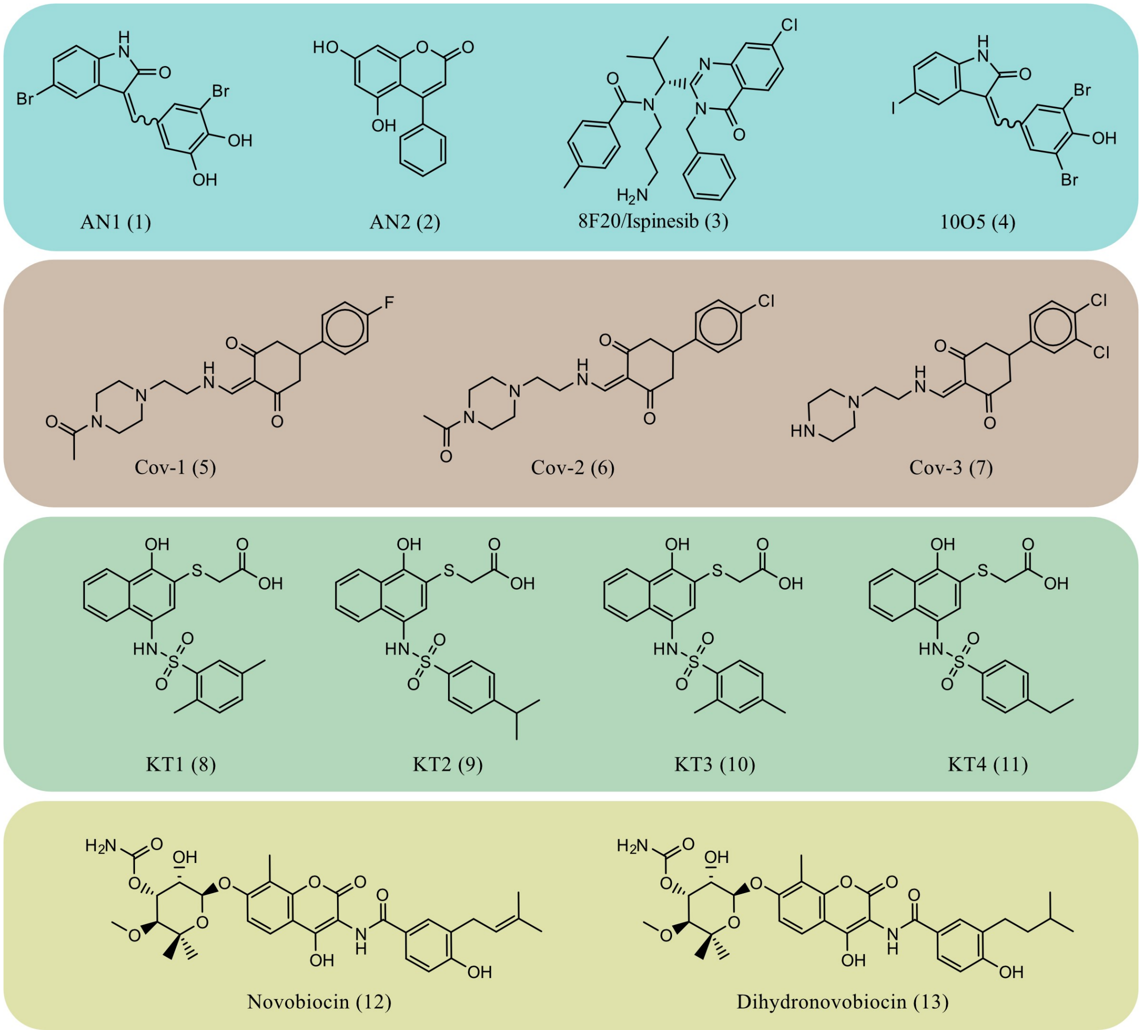

Supplementary Fig. 1: **Chemical structures of the compounds used in this study.** Cyan panel: prototypical ATTEC compounds published by Li et al.[1] Brown panel: Derivatives of covalent inhibitors reported by Fan et al.[2] Green panel: Derivatives of compounds reported by Tsuganezawa et al.[3] Yellow panel: Novobiocin and derivative reported by Hartmann et al.[4]

a

ΔTmB [°C]	p62-LIR	NIX-P2-LIR	AN1 (1)	AN2 (2)	8F20 (3)	10O5 (4)	Novobiocin (12)
LC3A	1.15 ± 0.04	2.39 ± 0.06	0.71 ± 0.23	0.63 ± 0.09	-0.43 ± 0.03	0.01 ± 0.20	0.80 ± 0.30
LC3B	1.00 ± 0.06	1.8 ± 0.17	0.69 ± 0.05	0.68 ± 0.22	-0.63 ± 0.06	0.43 ± 0.19	0.10 ± 0.66
LC3C	2.09 ± 0.15	4.35 ± 0.53	0.83 ± 0.21	0.33 ± 0.35	-0.41 ± 0.06	0.88 ± 0.29	0.03 ± 0.25
GABARAP	1.31 ± 0.18	3.12 ± 0.21	0.88 ± 0.10	0.04 ± 0.03	-0.72 ± 0.04	0.53 ± 0.16	0.13 ± 0.12
GABARAPL1	1.18 ± 0.11	3.41 ± 0.32	0.87 ± 0.21	0.83 ± 0.40	0.00 ± 0.34	1.62 ± 0.45	0.53 ± 0.15
GABARAPL2	0.92 ± 0.07	3.56 ± 0.07	0.77 ± 0.53	0.32 ± 0.07	-0.65 ± 0.41	1.32 ± 0.91	0.30 ± 0.10

b

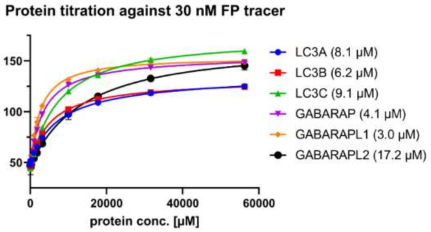

K_i [µM]	LC3A	LC3B	LC3C	GABARAP	GABARAPL1	GABARAPL2
p62-LIR	15.6	10.4	26.1	6.9	5.5	32.9
NIX-P2-LIR	9.7	16.1	7.5	4.9	3.7	6.3
LIR28	503.1	852.9	197.7	1.9	1.9	5.6
PCM1-LIR	179.0	299.0	84.3	10.1	11.2	26.2
AMBRA1-LIR	1003.8	1199.8	345.6	164.3	163.4	611.1

c

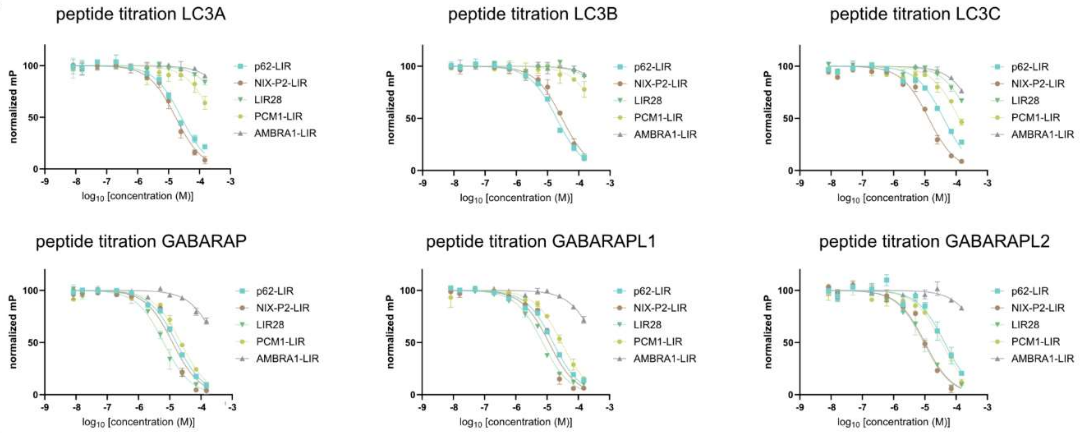

d

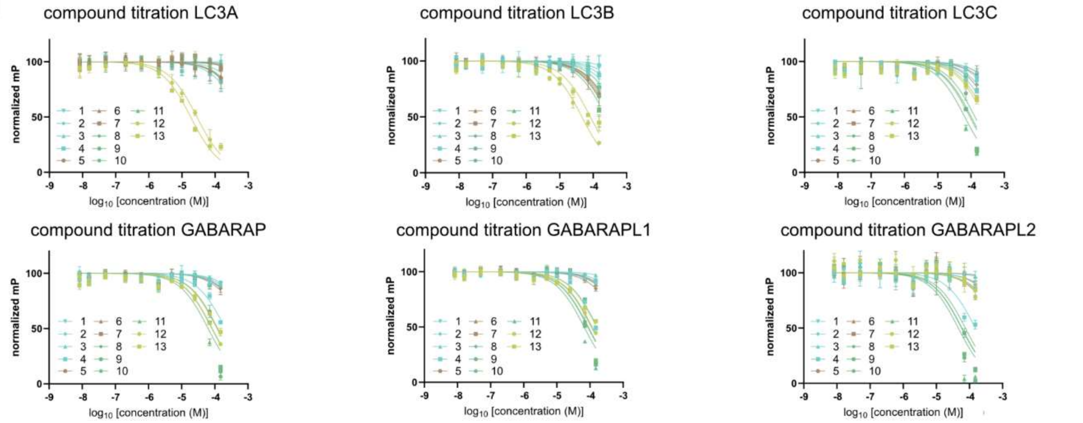

Supplementary Fig. 2: **Establishment of diverse binding assays for all human Atg8 homologs (LC3/GABARAPs). a** Results from DSF screening given as °C difference (according to Boltzmann fit) to the DMSO control (ΔTmB). Control peptides p62-LIR and NIX-P2-LIR stabilized the protein up to 4 °C, while compound-induced stabilization could not be observed, also for the positive control compound Novobiocin. Data were measured in technical replicates (n=3). **b** Protein titration of the different LC3/GABARAPs against the Cy5-

labelled p62-LIR peptide depicted with the calculated KD value (left). Table with measured Ki values for a set of different peptides (right), with corresponding curves shown in C). Data were measured in technical replicates (n=3) with error bars depicting the SD. Measured affinities are in agreement with the published affinity data.[5-9] **c** Peptide displacement experiments of five control peptides against all LC3/GABARAPs. Data were measured in technical replicates (n=2) with error bars depicting the SD. **d** Displacement curves of the compound set (**1-13**, Supplementary Fig. 1) against all LC3/GABARAPs measured in technical replicates (n=2) with error bars depicting the SD. Source data for Supplementary Fig. 2b,c,d are provided as a Source Data file.

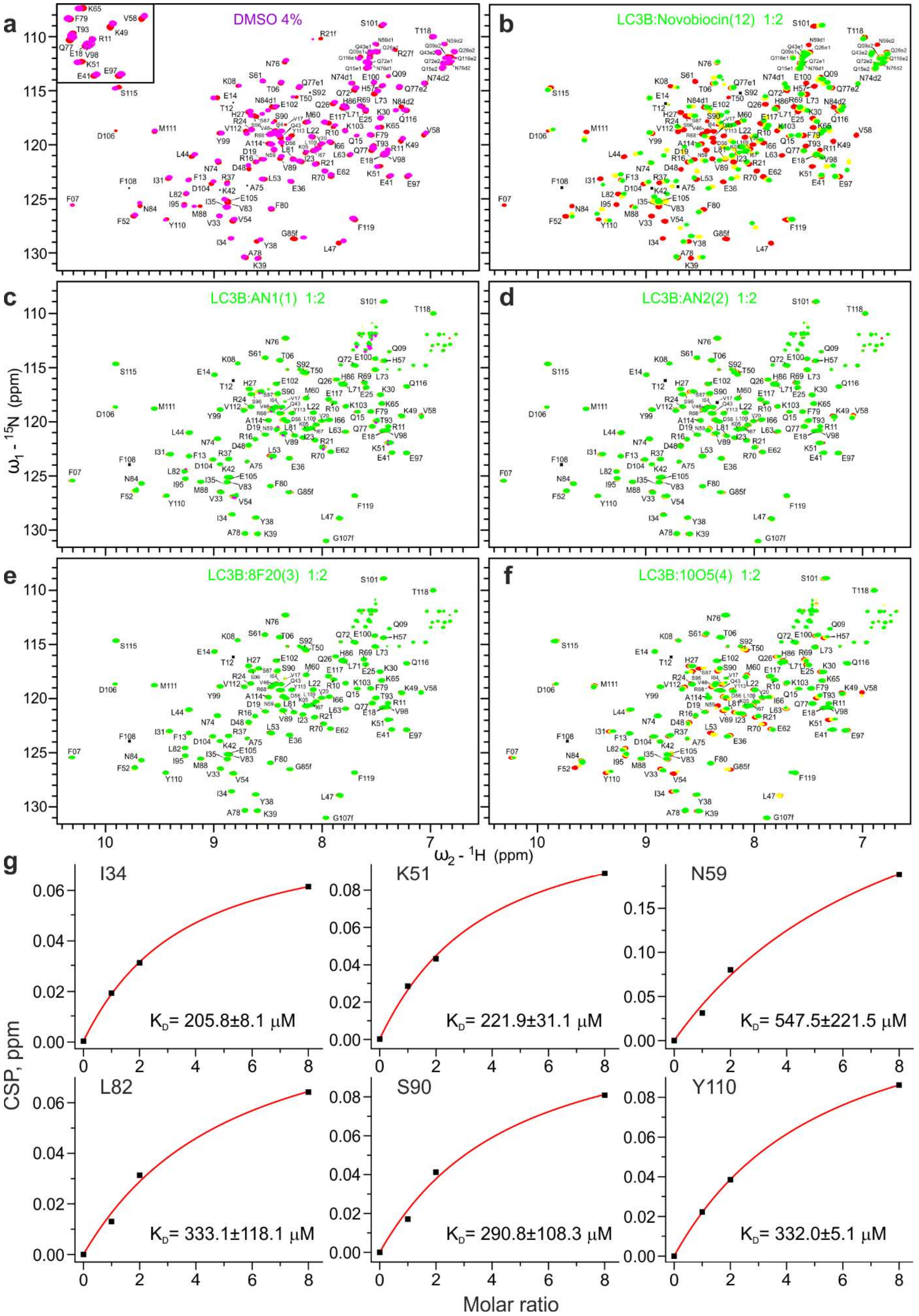

Supplementary Fig. 3: **Interaction between LC3B and compounds 12 and 1-4 investigated by NMR. a** CSPs of LC3B resonances induced by addition of 4% DMSO into ^{15}N-labelled

LC3B. Reference LC3B [^{1}H-^{15}N]-fHSQC spectrum (red) in overlay with the LC3B spectrum in presence of 4% DMSO (magenta). Both spectra were recorded at 700 MHz spectrometer. The small square in the upper left corner shows the LC3B representative (fingerprint) region around residues K51 and V58 depicted in Fig. 2d. **b** NMR titration of LC3B with positive control compound Novobiocin (**12**). Full [^{1}H-^{15}N]-HSQC spectra of free LC3B (red) overlaid with LC3B spectra in presence of Novobiocin (**12**) in 1:1 molar ratio (yellow) and 1:2 molar ratio (green). The spectra were recorded at 700 MHz spectrometer. **c-e** NMR titrations of LC3B with AN1 (**1**), AN2 (**2**) and 8F20 (**3**) in 1:1 molar ratio (yellow) and 1:2 molar ratio (green). The [^{15}N,^{1}H] BEST-TROSY spectra were recorded at 800 MHz spectrometer. **f** NMR titration of LC3B with 10O5 (**4**) in 1:1 molar ratio (yellow) and 1:2 molar ratio (green). The [^{15}N,^{1}H] BEST-TROSY spectra were recorded at 800 MHz spectrometer. **g** Estimation of K_D values for LC3B:10O5 interaction. K_D values (in μM) calculated for the selected LC3B residues (indicated on each plot). Original CSP values are shown as black squares, and the resulting fit is given as a red line in each plot. The selection criteria were I) the residues should be located within or near the interaction surfaces, II) the resonances should be clearly trackable at all titration steps. For all NMR experiments, n=1. Source data for Supplementary Fig. 3g are provided as a Source Data file.

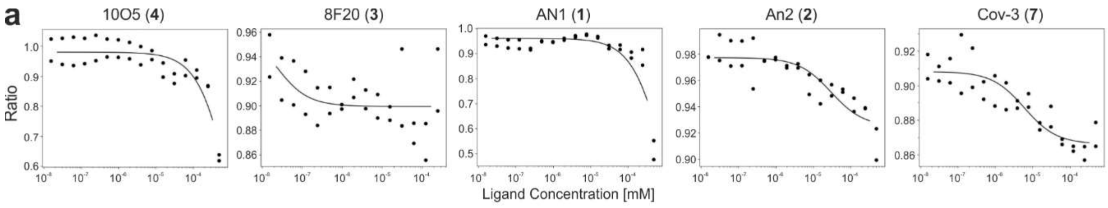

Ligand	Replicate (n)	Category	Saturation (%)	Δ Ratio	S/N	K_D
10O5 (**4**)	2	Potential Binder	30.9	-0.979	22.9	–
8F20 (**3**)	2	Non-binder	100.0	-0.064	3.1	–
An1 (**1**)	2	Potential Binder	34.2	-0.960	18.9	–
AN2 (**2**)	2	Non-binder	97.5	-0.040	4.3	–
Cov-3 (**7**)	2	Binder	84.8	-0.512	10.4	6.08

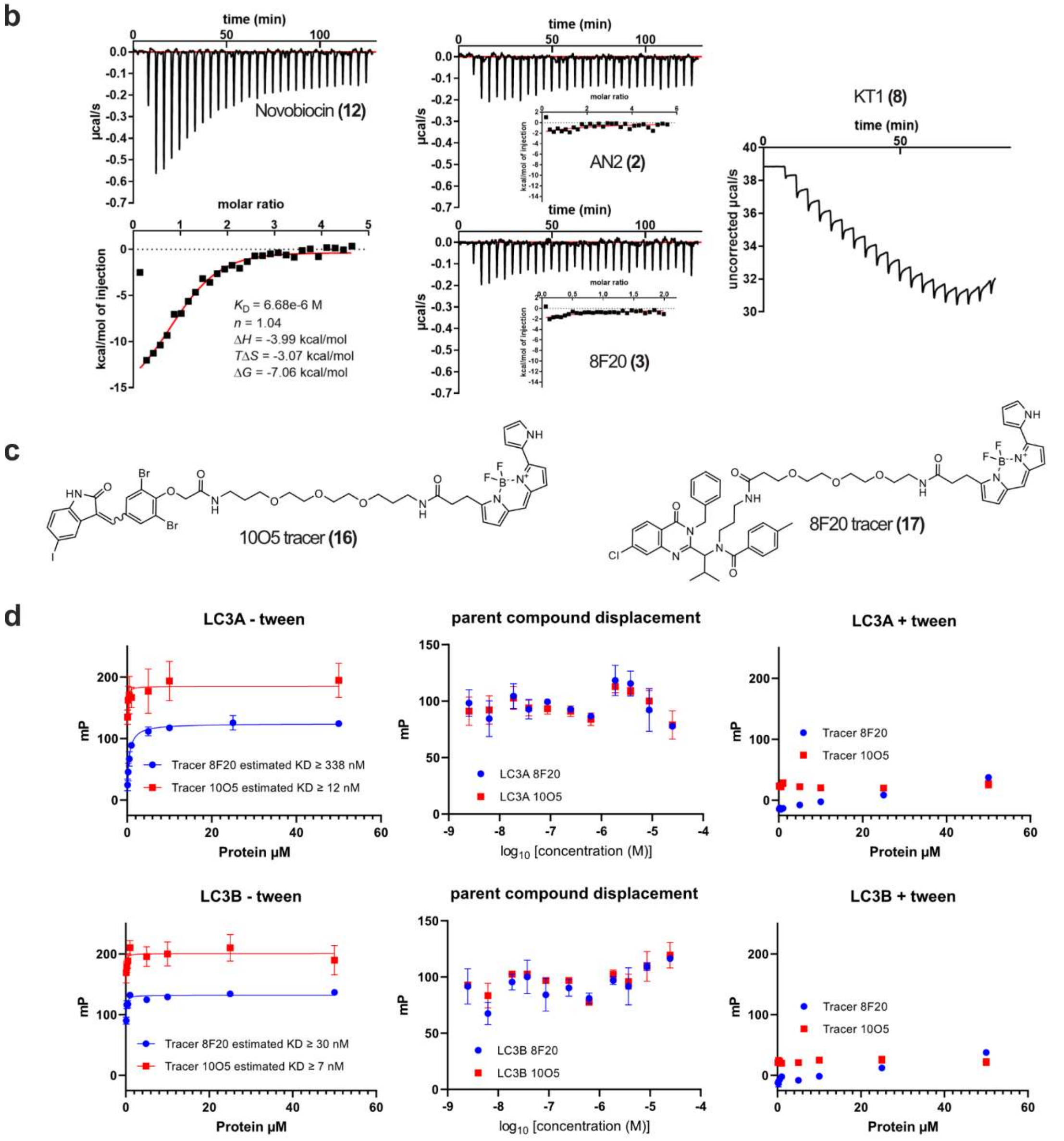

Supplementary Fig. 4: **Characterization of compounds binding to LC3/GABARAP proteins. a** Obtained plots from the spectral shift measurements of selected compounds (upper

panel) and the extracted binding information (lower table) (n=2)**.** **b** ITC measurement of Novobiocin (left measurement) and the ATTEC handles AN2 **(2)** and 8F20 **(3)** (middle panels) against LC3A. Other compounds (**1**, **4**, **5-8** etc.) displayed low solubility in the buffer system used and/or caused protein precipitation. These properties resulted in inconclusive ITC measurements as exemplary depicted for compound **8** (right upper panel) (n=1). **c** Structure of 10O5 (**4**)-based tracer compound **16** and the 8F20 (**3**)-based tracer compound **17**. **d** Fluorescence polarization assay utilizing tracers **16** and **17** for interaction studies with LC3A (upper panels) and LC3B (lower panels). The left panels depict protein titration measurements for tracer K_D determination indicating nanomolar tracer affinity as presented in[10], lacking detergent in the buffer to suppress unspecific binding. Middle panels depict displacement experiments using compound **3** for tracer **17** and compound **4** for tracer **16** showing no displacement, indicating unspecific binding during protein titration. Right panels show a repeated protein titration with 0.05% Tween20 supplemented buffer, completely abolishing the putative binding, revealing unspecific binding of the tracers. Data were collected in technical triplicates with error bars expressing the SD (n=3). Source data for Supplementary Fig. 4a,b,d are provided as a Source Data file.

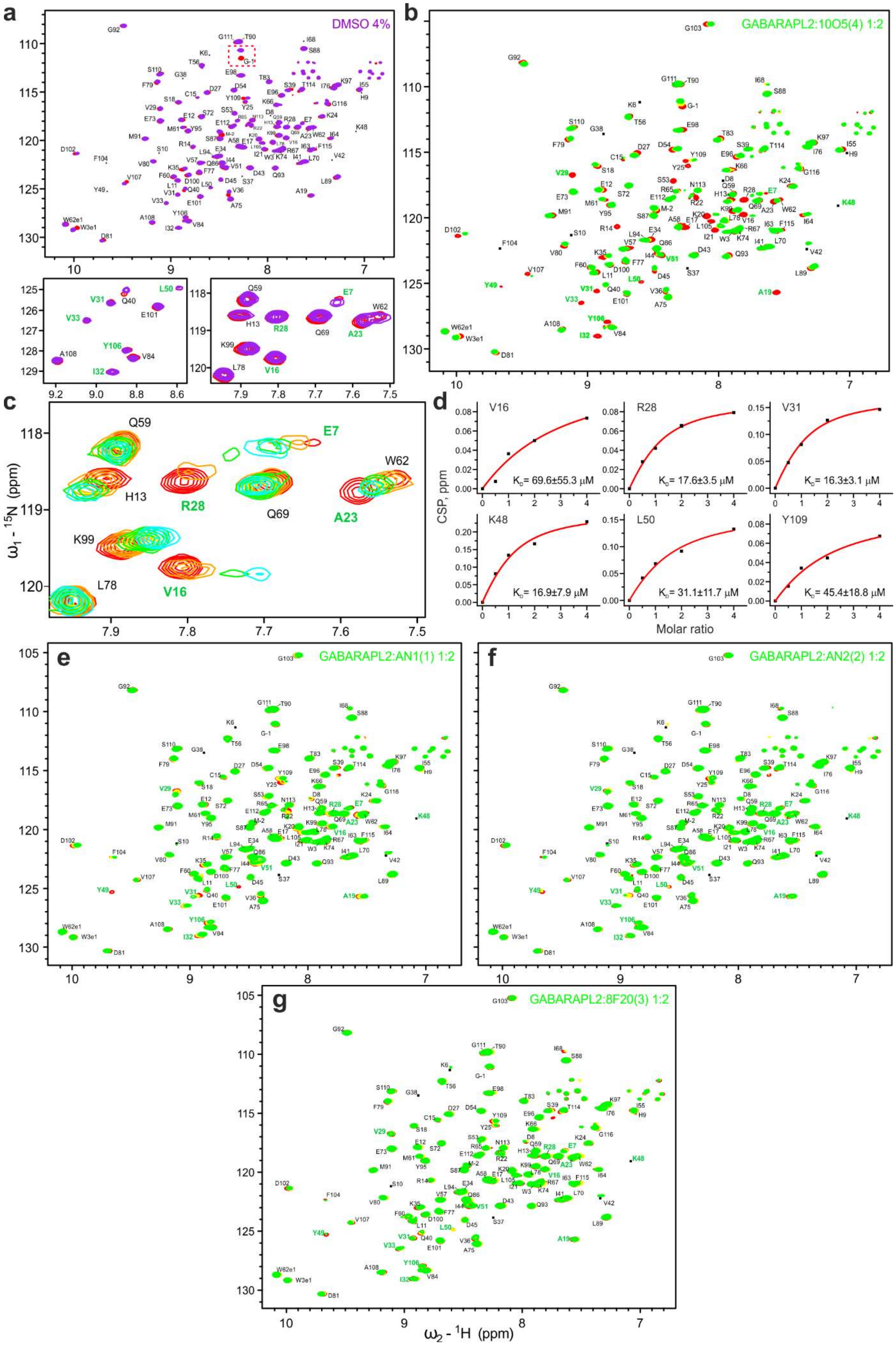
a
DMSO 4%
b
GABARAPL2:10O5(4) 1:2
c
ω1 - 15N (ppm)
d
V16
K_D = 69.6±55.3 μM
R28
K_D = 17.6±3.5 μM
V31
K_D = 16.3±3.1 μM
K48
K_D = 16.9±7.9 μM
L50
K_D = 31.1±11.7 μM
Y109
K_D = 45.4±18.8 μM
CSP, ppm
Molar ratio
e
GABARAPL2:AN1(1) 1:2
f
GABARAPL2:AN2(2) 1:2
g
GABARAPL2:8F20(3) 1:2
ω2 - 1H (ppm)

Supplementary Fig. 5: **Interaction between GABARAPL2 and compounds 1-4 investigated by NMR. a** CSPs of GABARAPL2 resonances induced by addition of 4% DMSO into ^{13}C,^{15}N-labelled GABARAPL2. Upper plot: reference GABARAPL2 [^{15}N,^{1}H] BEST-TROSY (900 MHz spectrometer) full spectrum (red) in overlay with the GABARAPL2 spectrum in presence of 4% DMSO. Lower plots show the GABARAPL2 representative (fingerprint) regions around residues L50 (left) and V16 (right). **b-e** NMR titration of GABARAPL2 with 10O5 (**4**). **b** Full [^{15}N,^{1}H] BEST-TROSY spectrum (900 MHz spectrometer) of free GABARAPL2 (red) overlaid with GABARAPL2 spectrum in presence of 10O5 in 1:6 molar ratio (blue). **c** Representative region of the [^{15}N,^{1}H] BEST-TROSY GABARAPL2 spectrum upon titration of GABARAPL2 with 10O5 (free protein – red, molar ratios of 1:0.5, 1:1, 1:2 and 1:4 are given in orange, yellow, green and cyan, respectively). **d** Estimation of K_D values for GABARAPL2:10O5 (**4**) interaction. K_D values (in μM) calculated for the selected GABARAPL2 residues (indicated on each plot). Original CSP values are shown as black squares, and the resulting fit is given as a red line in each plot. The selection criteria were identical to the one described in Supplementary Fig. 3. **e-g** NMR titrations of GABARAPL2 with AN1 (**1**), AN2 (**2**) and 8F20 (**3**), respectively (600 MHz spectrometer). For all NMR experiments, n=1. Source data for Supplementary Fig. 5d are provided as a Source Data file.

a

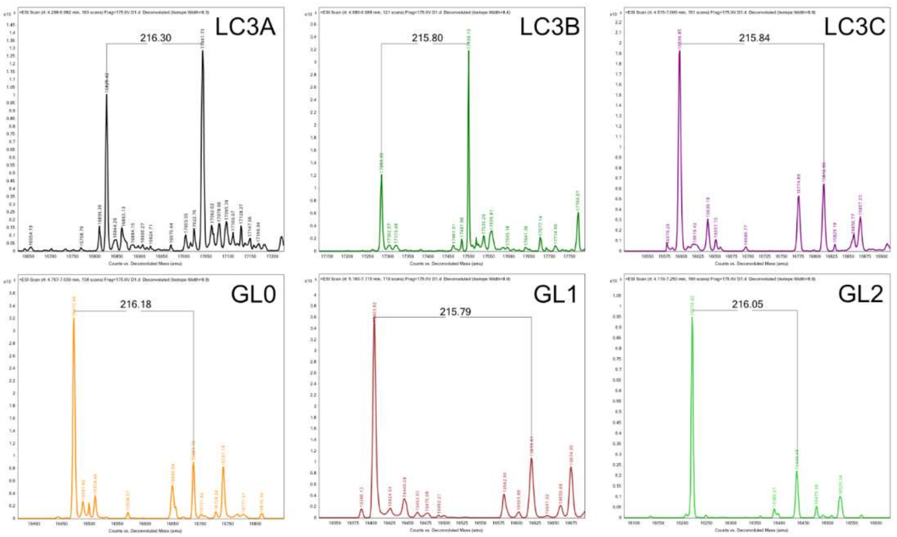

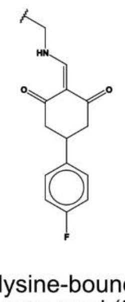

lysine-bound compound (5)

b

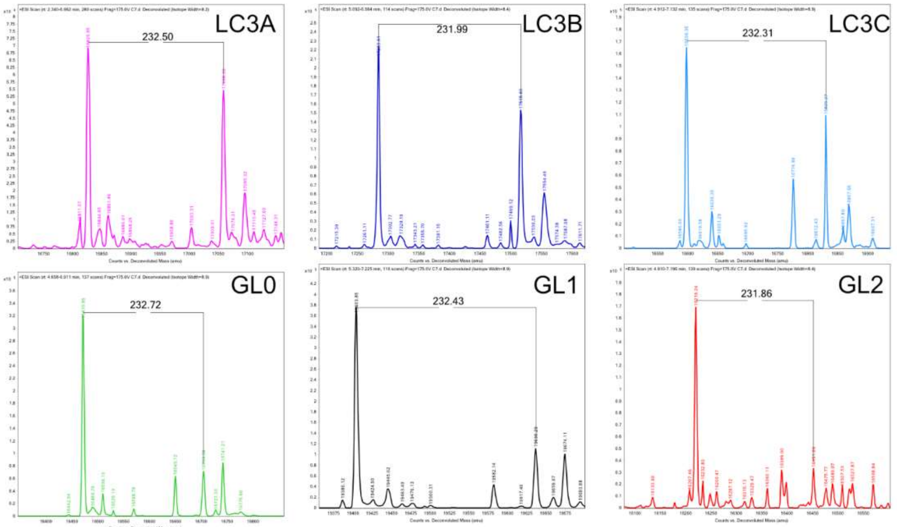

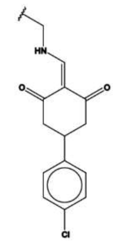

lysine-bound compound (6)

c

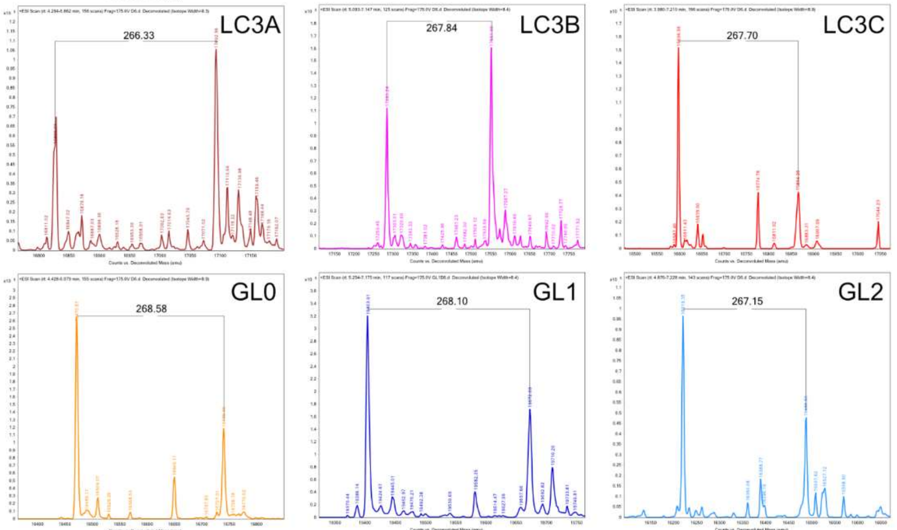

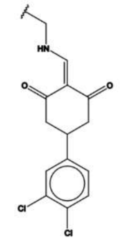

lysine-bound compound (7)

Supplementary Fig. 6: **ESI mass spectrometry for all LC3/GABARAP proteins treated with compounds 5-7**. **a** Mass shifts obtained from treatment with compound **5**. All LC3/GABARAPs were successfully labelled with the highest labelling ratio on LC3A und LC3B. **b** Mass shifts obtained from treatment with compound **6**. All LC3/GABARAPs were successfully labelled with the highest labelling ratio of all LC3 proteins. **c** Mass shifts obtained from treatment with compound **7**. All Atg8 homologs were successfully modified with the highest ratio on LC3A und LC3B (n=1)

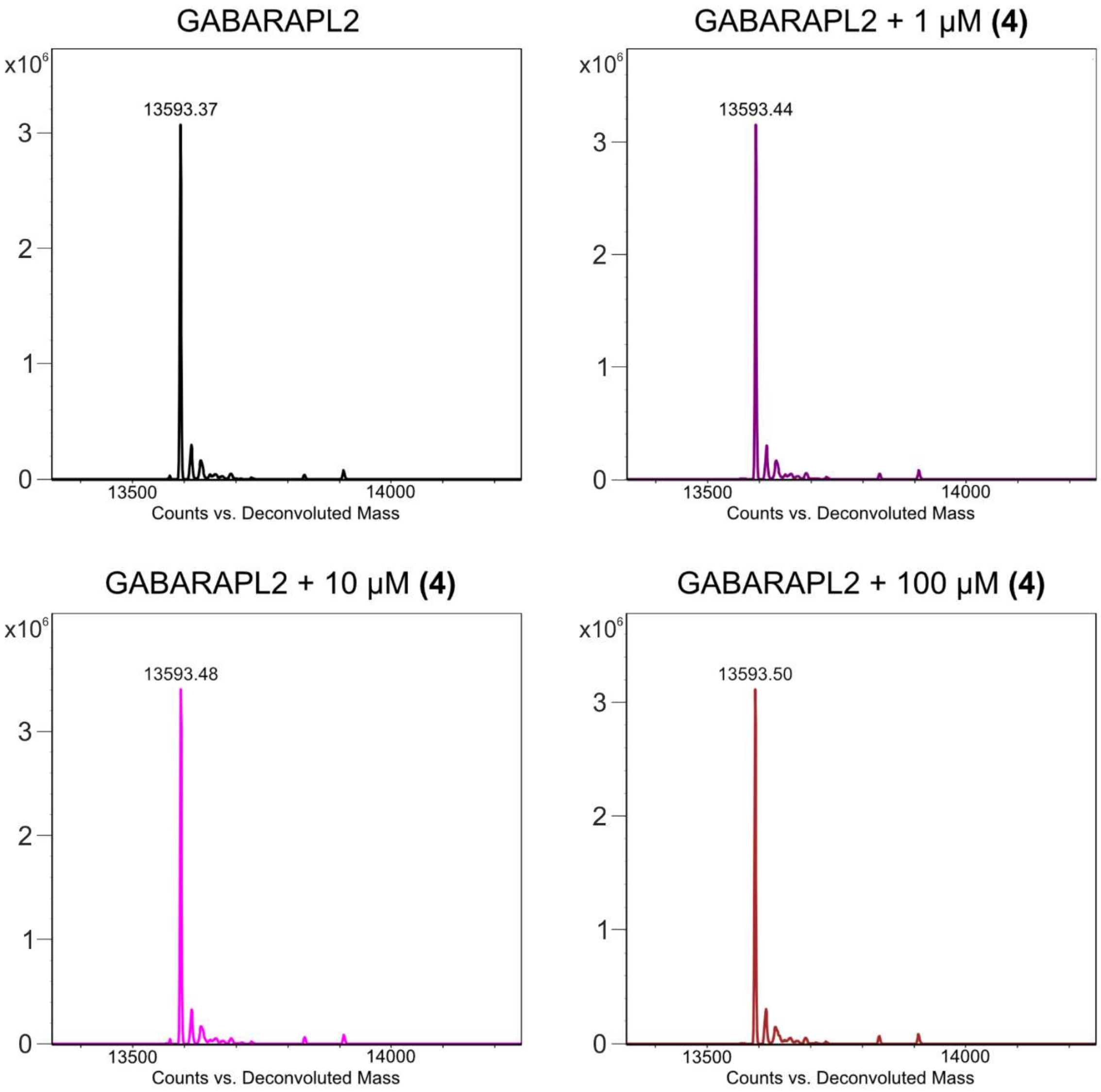

Supplementary Fig. 7: **ESI mass spectrometry for GABARAPL2 treated with different concentrations of compound 4**. To test covalent modification of GABARAPL2 (targeted by **4** with the highest affinity) different concentrations of 4 were incubated for 90 minutes at room temperature with 50 µM protein. None of the treatments showed a mass shift (as seen e.g. in Supplementary Figure 6) indicating no covalent modification of GABARAPL2 by 10O5 (n=1).

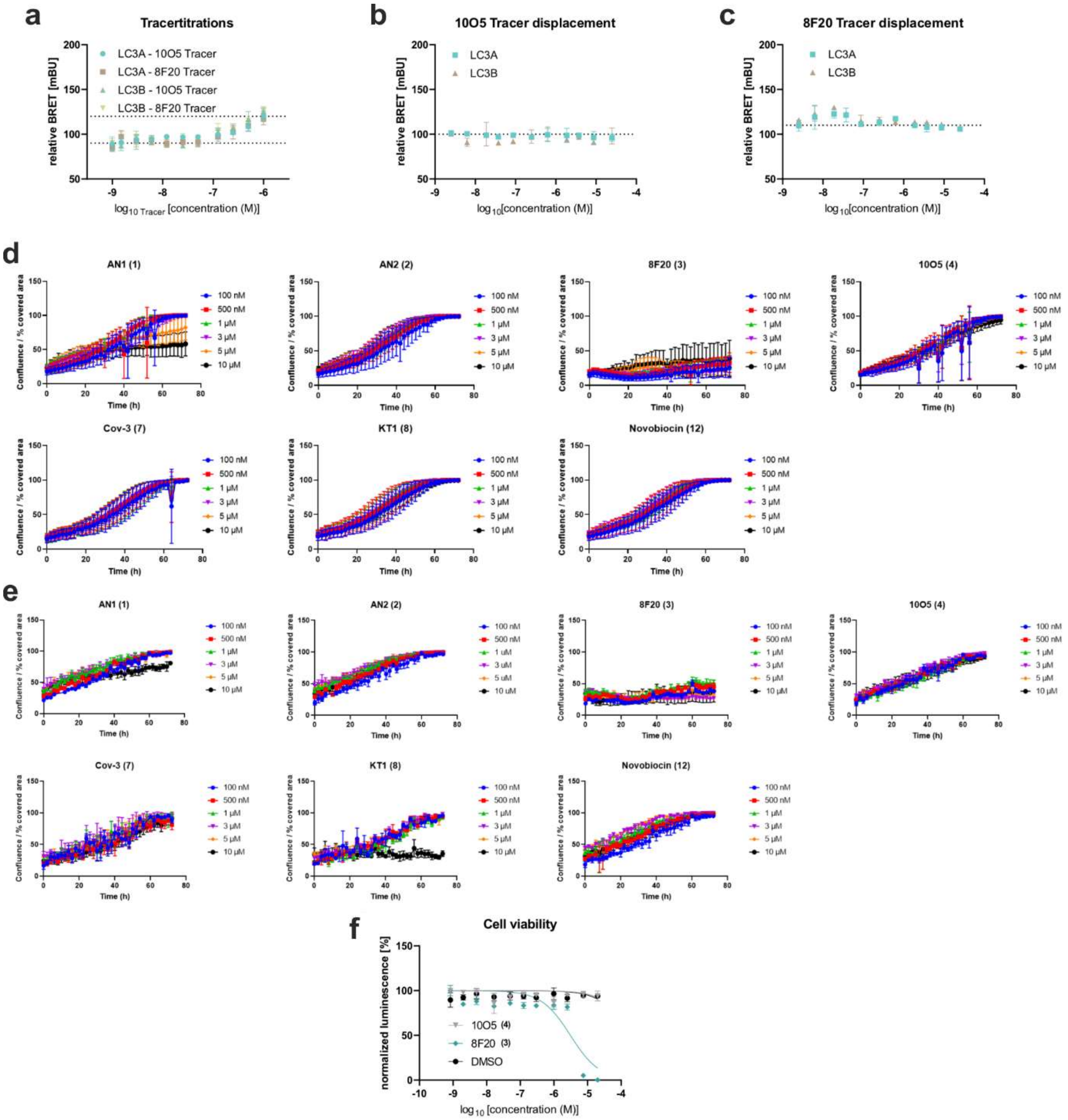

Supplementary Fig. 8: **Cellular assays. a** tracer titration of compounds **16** and **17** (Supplementary Fig. 4) against NanoLuciferase-tagged LC3A and LC3B showing a maximum BRET increase of ~1.3-fold, indicating unspecific BRET increase due to the presence of higher fluorophore concentrations. Displacement assays were carried out to test specific compound binding on LC3A (**b**) and LC3B (**c**) through tracer displacement using compound **4** for tracer compound **16** and compound **3** for tracer compound **17**. No displacement at 1 µM of the tracer compounds proves the hypothesis of unspecific BRET increase, therefore showing no compound-LC3 interaction. Measurements were carried out in biological replicates (n=2) with

error bars expressing the SD. **d** RPE1 growth was detected based on the cells confluence within the well. Outliers were determined to be faulty collected images. Apart from high dosage effects of compounds **1**, only compound **3** lead to significant growth reduction. Data were collected in biological replicates with technical triplicates each (n=6) and are presented as a means with error bars expressing the SD. **e** Growth curves of U2OS cells, treated with different small molecules. Growth was detected based on the cell confluence in the well. For U2OS cells, compound **8** shows a drastic effect at the highest concentration, while compound **3** leads to significant growth inhibition in all tested concentrations. Data was collected in technical triplicates (n=3) and error bars expressing the SD. **f** Cell viability measurements of the ATTEC handles 8F20 (**3**) and 10O5 (**4**) indicating no viability/toxicity effect of compound **4** while compound **3** treatment led to viability loss in the high μM range. Data were collected in biological replicates (n=2) with error bars expressing the SD. Data agrees with published viability data.[1] Source data for Supplementary Fig. 8a-f are provided as a Source Data file.

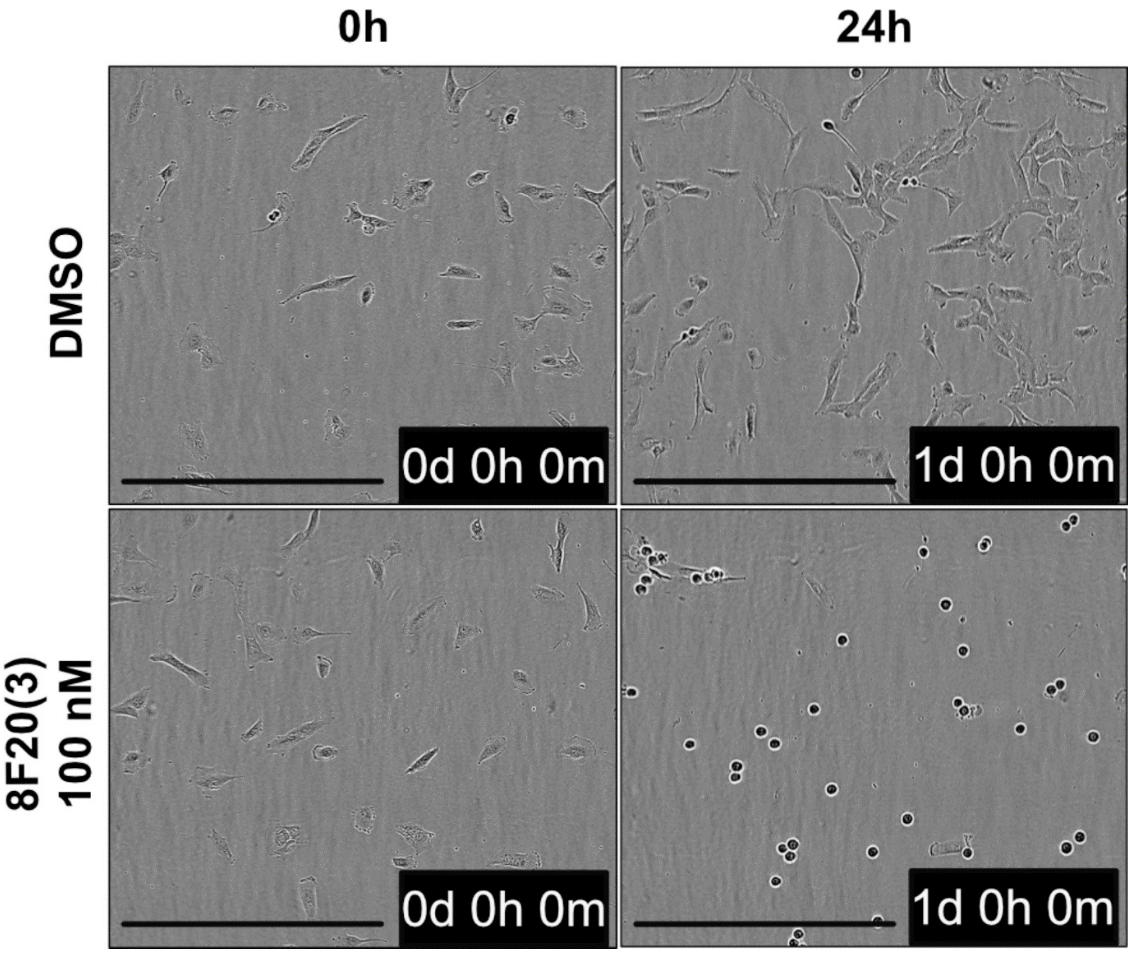

Supplementary Fig. 9: **Microscopy images** acquired with the IncuCyte S3 (10x) show rounding of RPE1 cells after treatment with compound **3** for 24h. Scale bar reflects 500 um.

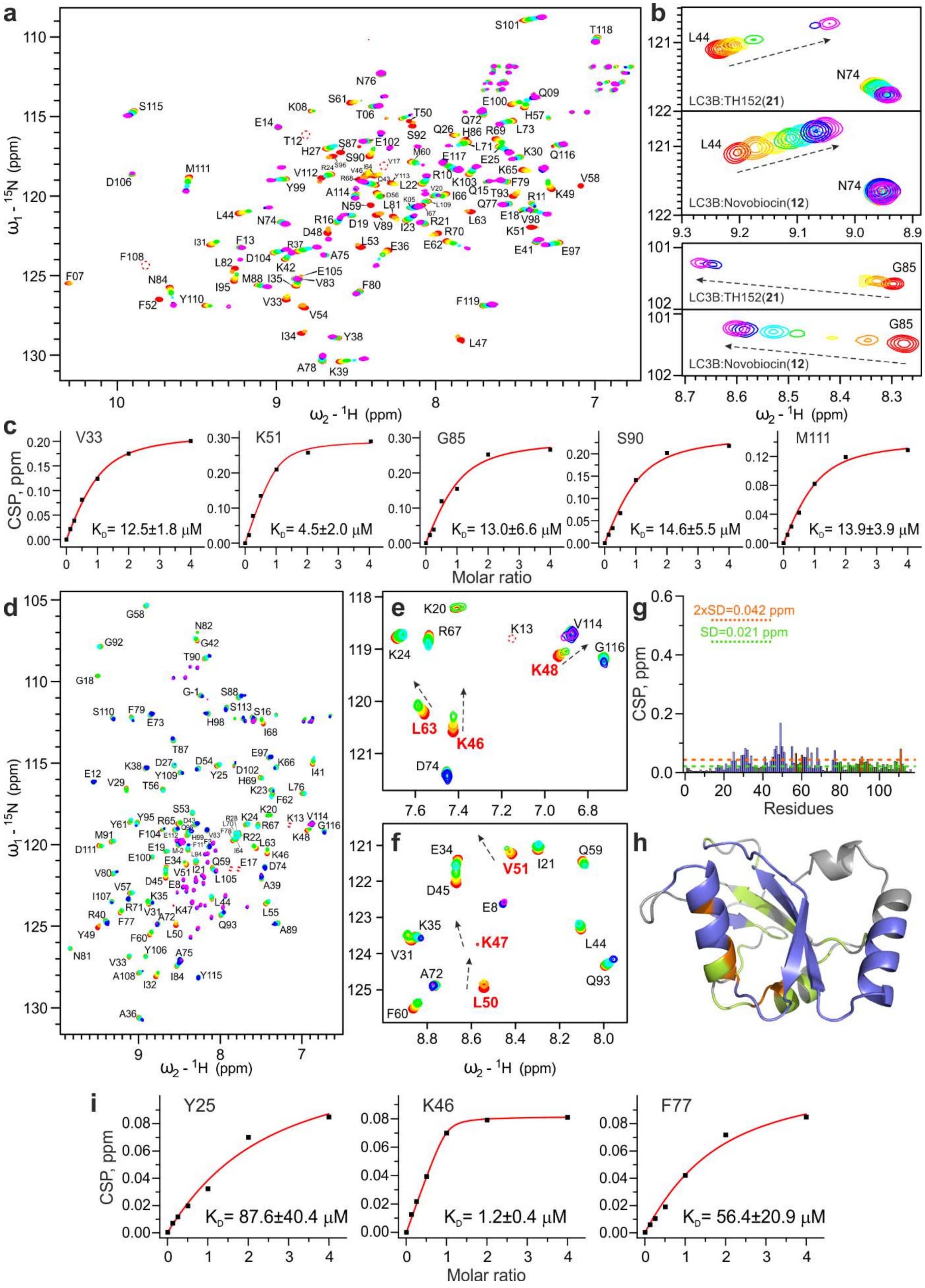

Supplementary Fig. 10: **Interaction between LC3B/GABARAP proteins and compound 21. a-d** Interaction between LC3B and compound TH152 (**21**) investigated by NMR. **a** Full [^{15}N,^{1}H] BEST-TROSY spectrum (950 MHz spectrometer) of free LC3B (red) overlaid with

LC3B spectra in presence of **21** in increasing molar ratios (1:0.125 – orange, 1:0.25 – yellow, 1:0:5 – green, 1:1 – cyan, 1:2 – blue and 1:4 - magenta). **b** LC3B:**21** interaction revealed strong similarity with LC3B:**12** interaction. Upper plot: representative spectra area around L44 backbone HN resonance for titration with **21** and **12** (indicated on each graph). Lower plot: representative spectra area around G85 backbone HN resonance for titration with **21** and **12**. **c** Estimation of K_D values for LC3B:**21** interaction. K_D values calculated for the selected LC3B residues (indicated on each plot) upon titration with **21**. Original CSP values are shown as black squares and the resulting fit is given as a red line in each plot. The selection criteria were identical as described in Supplementary Fig. 3. **d-i** Interaction between GABARAP and compound **21** investigated by NMR. **d** Full [^{15}N,^{1}H] BEST-TROSY spectrum (950 MHz spectrometer) of free GABARAP overlaid with GABARAP spectra in presence of **21** in increasing molar ratios (the same colorcode as for LC3B titration). Non-assigned peaks in the presented spectra are from Gln and Asn sidechain NH_2 resonances, and from traces of the GABARAP degradation peptides. **e-f** Representative regions of the [^{15}N,^{1}H] BEST-TROSY GABARAP spectra around key residues K46, K48 and L63 (**e**), and K47, L50 and V51 (**f**) upon titration of GABARAP with **21** (the same colorcode as above). **g** CSP values, induced by compounds **21** at molar ratio 1:2, are plotted against GABARAP residue numbers. The light green dashed line indicates the standard deviations (SD) over all residues, the orange dashed line indicates double SD values. Residues with small (CSP < SD), intermediate (SD < CSP < 2xSD) or strong (2xSD < CSP) CSP values are marked in grey, light green and orange, respectively. GABARAP residues which undergo strong intermediate exchange mode (significant decrease of the resonances intensity upon titration with **21**) are marked blue. **h** 3D mapping of CSP values on GABARAP structure (PDB ID 1UGM), indicating both hydrophobic pockets, HP1 and HP2, as most relevant interaction sites. The colorcode is the same as above. **i** Estimation of K_D values for GABARAP:**21** interaction. K_D values (in μM) calculated for the selected GABARAP residues (indicated on each plot) upon titration with **21**. Original CSP values are shown as black squares, and the resulting fit is given as a red line in each plot. The selection criteria were identical as described in Supplementary Fig. 3. For all NMR experiments, n=1. Source data for Supplementary Fig. 10c,g,i are provided as a Source Data file.

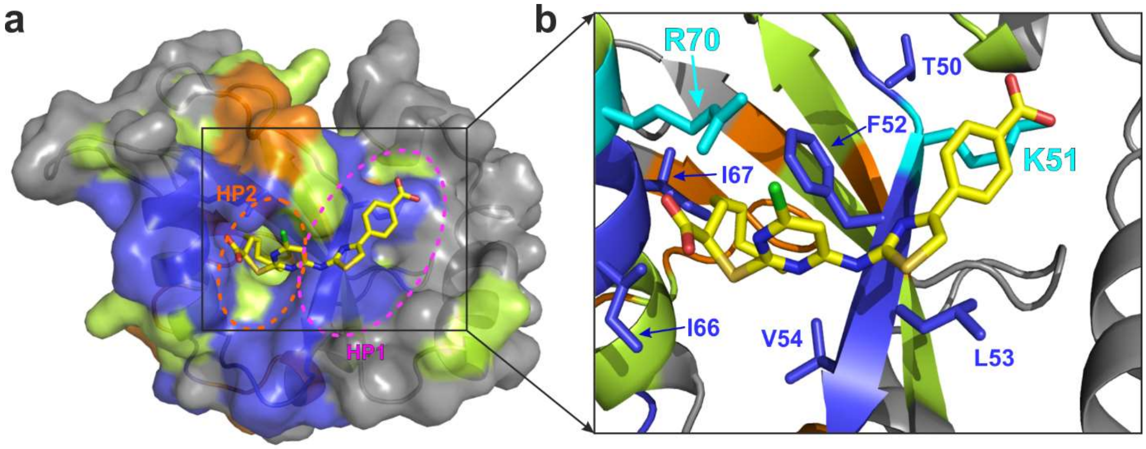

Supplementary Fig. 11: **Results of TH152 (21) docking onto the LC3A structure with interacting residues marked as determined by NMR. a** Surface representation of the LC3A structure (PDB ID 6TBE, the same orientation as in Fig. 4e,f) with residues marked by NMR-evaluated strength of interaction observed for LC3B, the closest homolog of LC3A. Residues with small (CSP < SD), intermediate (SD < CSP < 2xSD) or strong (2xSD < CSP) CSP values are marked in grey, light green and orange, respectively. LC3B residues which undergo strong intermediate exchange mode are marked blue. The structure of TH152 is shown as yellow sticks. Relative positions of hydrophobic pockets HP1 (magenta) and HP2 (orange) are shown by dashed lines. **b** TH152 docked to LC3A (detailed view). Most affected LC3A residues are shown as blue sticks (according to the NMR results for LC3B), key residues K51 and R70 are marked cyan.

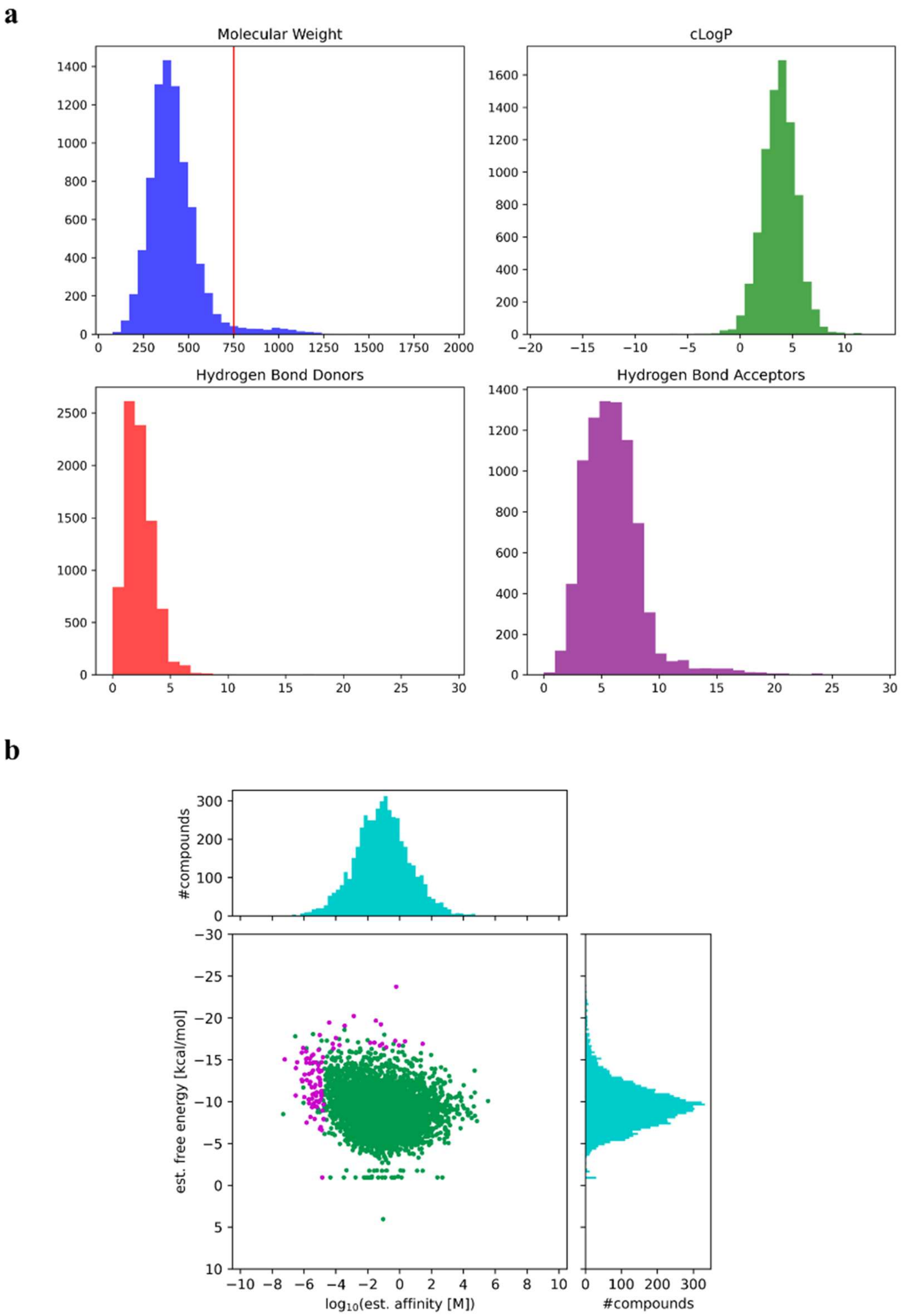

Supplementary Fig. 12: **Chemical properties of virtually screened compounds and distribution of resulting scores. a** Chemical properties of the compounds from the in-house library were calculated using the RDKit library. The vertical red line indicates the 750 Da molecular weight threshold, which has been chosen to obtain a set of hits that exhibit drug-like features. **b** Distributions of resulting docking scores from the two docking platforms employed

before applying the molecular weight cut-off. Pink coloration indicates compounds that were selected for the follow up experiments. Source data for Supplementary Fig. 12a,b are provided as a Source Data file.

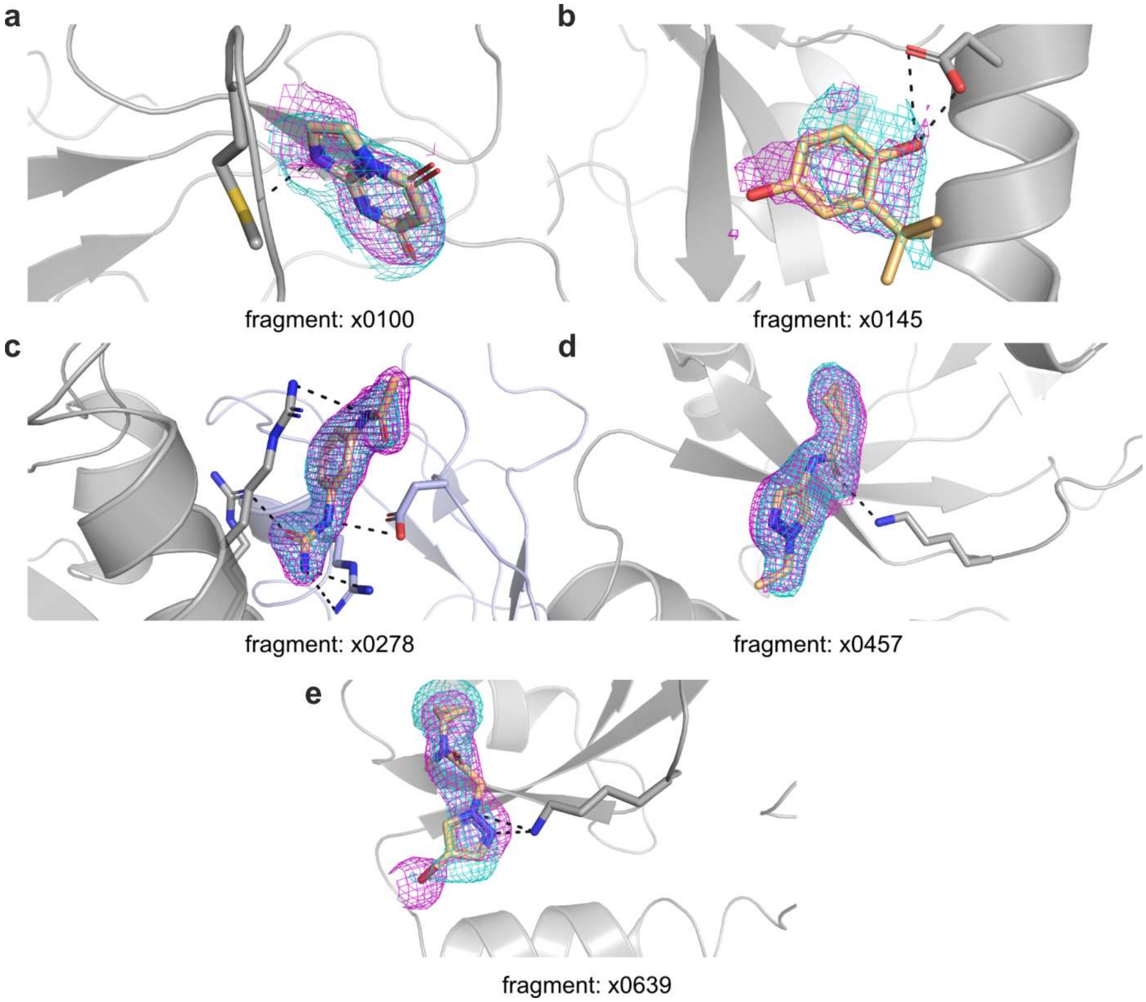

Supplementary Fig. 13: **Electron densities.** Crystallized fragments (**a-e**, each fragment is indicated) bound to LC3B as depicted in Fig. 5. Together with the fragments bound in the crystal structure, the event map (cyan) and the 2Fo-Fc (magenta) are depicted together with interacting residues.

Supplementary Table 1: **Structure-activity relationship of sulfonamide-based compounds[11-13] on LC3A tracer displacement, normalized on p62-LIR positive control.**

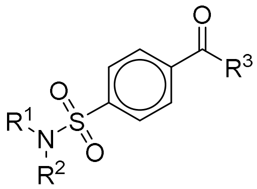

Cpd-ID	R^1	R^2	R^3	% tracer bound @LC3A
(TH061)	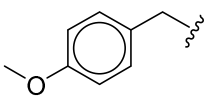	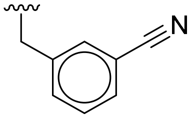	OH	83
(TH119)	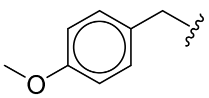	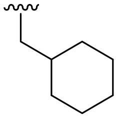	OH	92
(TH133)	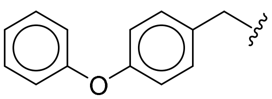	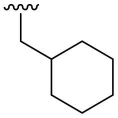	OH	82
(TH253)	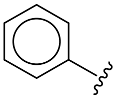	H	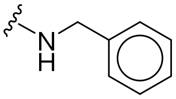	88
(TH676)	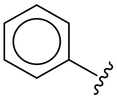	H	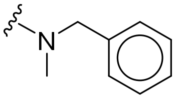	84
(TH281)	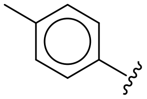	H	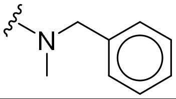	95
(TH677)	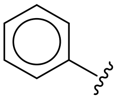	H	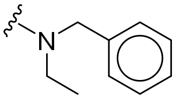	87

Supplementary Table 2: **Structure-activity relationship of benzothiazole based compounds[11,14] on LC3A tracer displacement, normalized on p62-LIR positive control.**

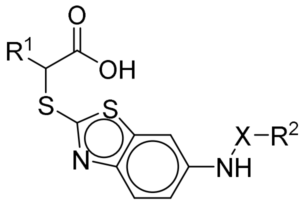

Cpd-ID	R^1	R^2	X	% tracer bound @LC3A
(TH195)	*n*-hexyl	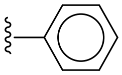	CH_2	78
(TH185)	*n*-hexyl	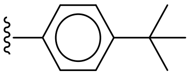	CH_2	82
(TH198)	*n*-hexyl	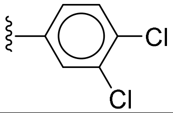	CH_2	79
(TH191)	*n*-hexyl	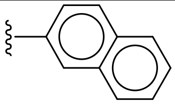	CH_2	78
(TH200)	*n*-hexyl	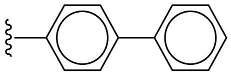	CH_2	72
(TH181)	*n*-hexyl	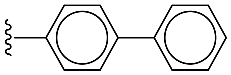	C=O	78
(TH177)	*n*-hexyl	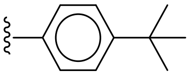	C=O	85
(TH188)	*n*-butyl	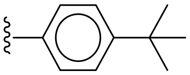	C=O	89

Supplementary Table 3: **Structure-activity relationship of pyrimidine based compounds[11,15] on LC3A tracer displacement, normalized on p62-LIR positive control.**

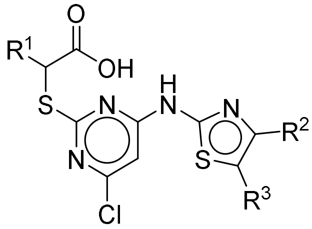

Cpd-ID	R^1	R^2	R^3	% tracer bound @LC3A
(TH020)	*n*-hexyl	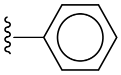	H	84
(TH018)	*n*-hexyl	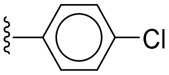	H	81
(TH035)	ethyl	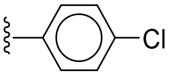	H	85
(TH026)	*n*-hexyl	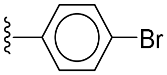	H	79
(TH057)	*n*-hexyl	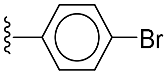	Methyl	94
(TH100)	*n*-hexyl	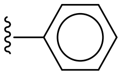	Ethyl	77
(TH083)	*n*-hexyl	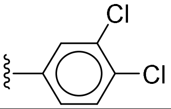	H	84
(TH102)	*n*-hexyl	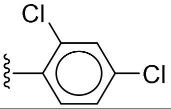	H	78
(TH044)	*n*-hexyl	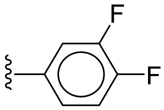	H	76
(TH054)	*n*-hexyl	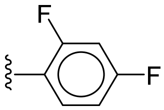	H	75
(TH084)	*n*-hexyl	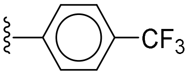	H	76
(TH107)	ethyl	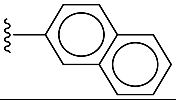	H	82
(TH034)	*n*-hexyl	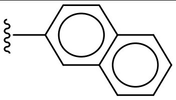	H	79
(TH104)	*n*-hexyl	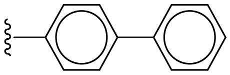	H	82

(TH105)	*n*-hexyl	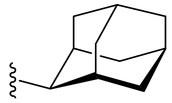	H	84
(TH062)	*n*-hexyl	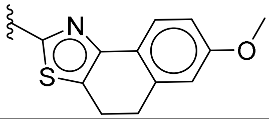		76
(TH121)	*n*-hexyl	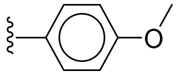	H	83
(TH056)	*n*-hexyl	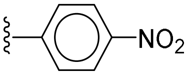	H	61
**21 (TH152)**	*n*-hexyl	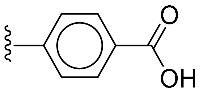	H	26

Supplementary Table 4: **X-ray data collection and refinement statistics**

	8Q53	7GAU	7GA8	7GA9	7GAA	7GAB
**Data collection**[a]						
Wavelength (Å)	0.99999	0.92124	0.92124	0.92124	0.92124	0.92124
Space group	P43	P43	P43	P43	P43	P43
Resolution (Å)	42.61 – 1.36	61.00 – 1.59	30.68 – 1.87	30.49 – 2.17	30.91 – 2.03	43.05 – 2.23
Last resolution shell (Å)	1.38 – 1.36	1.62 – 1.59	1.91 – 1.87	2.23 – 2.17	2.08 – 2.03	2.31 – 2.23
Unit cell parameters						
a,b,c (Å)	60.26, 60.26, 34.93	61.01, 61.01, 35.48	60.83, 60.83, 35.53	60.98, 60.98, 35.32	60.59, 60.59, 35.93	60.89, 60.89, 35.59
α, β, γ (°)	90, 90, 90	90, 90, 90	90, 90, 90	90, 90, 90	90, 90, 90	90, 90, 90
Total number of observations	181809 (17402)	241587 (10881)	150104 (9935)	93140 (6872)	116879 (8961)	87203 (7854)
Unique reflections	26382 (2575)	17826 (896)	10871 (687)	7021 (515)	8621 (641)	6512 (584)
Mosaicity (˚)	0.13	0	0	0	0	0
Multiplicity	6.9 (6.8)	13.6 (12.1)	13.8 (14.5)	13.3 (13.3)	13.6 (14.0)	13.4 (13.4)
Mean I/σ(I)	21.11 (2.40)	11.9 (0.3)	14.4 (0.2)	8.8 (0.9)	12.3 (0.4)	10.3 (0.5)
Completeness (%)	97.08 (96.12)	100.0 (100.0)	99.5 (100.0)	99.7 (98.4)	100.0 (100.0)	100.0 (100.0)
$R_{merge}{}^{b}$	0.042 (0.733)	0.092 (3.870)	0.083 (4.560)	0.199 (3.303)	0.186 (10.910)	0.144 (3.603)
$R_{meas}{}^{c}$	0.046 (0.794)	0.095 (1.040)	0.087 (4.725)	0.208 (3.433)	0.194 (11.319)	0.150 (3.747)
$R_{pim}{}^{d}$	0.017 (0.302)	0.026 (1.150)	0.024 (1.235)	0.058 (0.931)	0.053 (3.000)	0.041 (1.022)
**Refinement**						
Resolution (Å)	42.61 – 1.36	61.00 – 1.59	30.68 – 1.87	30.49 – 2.17	30.91-2.03	43.05 – 2.23
Reflections used	26378	16693	10093	6749	8268	6243
Free R flagged reflections	1934	780	426	266	330	251
$R_{cryst}{}^{e}$	0.190	0.193	0.216	0.180	0.190	0.187
$R_{free}{}^{f}$	0.210	0.215	0.274	0.246	0.248	0.276
rmsd bonds (Å)	0.007	0.008	0.006	0.008	0.007	0.009
rmsd angles (˚)	0.99	1.542	1.428	1.518	1.547	1.625
Ramachandran plot						
Most favored (%)	99.12	99.12	99.12	99.12	99.12	97.35
Additionally allowed (%)	0.88	0.88	0.88	0.88	0.88	2.65
Mean B-factor (Å^2)	25.830	36.989	55.917	56.978	65.188	66.317

[a]Values for the last resolution shell are in parentheses.

[b]$R_{merge} = \Sigma_{hkl}\Sigma_i|I_i(hkl)-\langle I(hkl)\rangle| / \Sigma_{hkl}\Sigma_i I_i(hkl)$, where I(hkl) is the intensity of reflection hkl

[c]$R_{meas} = \Sigma_{hkl}(n/(n-1))^{1/2}\Sigma_i|I_i(hkl)-\langle I(hkl)\rangle| / \Sigma_{hkl}\Sigma_i I_i(hkl)$

[d]$R_{pim} = \Sigma_{hkl}(1/(n-1))^{1/2}\Sigma_i|I_i(hkl)-\langle I(hkl)\rangle| / \Sigma_{hkl}\Sigma_i I_i(hkl)$

[e]$R_{cryst} = \Sigma_{hkl} ||F_{obs}|-|F_{calc}|| / \Sigma|F_{obs}|$

[f]R_{free} is the cross-validation R-factor computed for the test set of unique reflections.

	7GAC	7GAD	7GAE	7GAF	7GAG	7GAH
**Data collection**[a]						
Wavelength (Å)	0.92124	0.92124	0.92124	0.92124	0.92124	0.92124
Space group	P43	P43	P43	P43	P43	P43
Resolution (Å)	42.88 – 1.91	61.02 – 1.86	43.08 – 1.92	42.98 – 1.84	61.00 – 1.59	30.60 – 1.90
Last resolution shell (Å)	1.95 – 1.91	1.90 – 1.86	1.96 – 1.92	1.88 – 1.84	1.62 – 1.59	1.95 – 1.90
Unit cell parameters						
a,b,c (Å)	60.62, 60.62, 35.85	61.03, 61.03, 35.55	60.92, 60.92, 35.65	60.80, 60.80, 35.38	61.01, 61.01, 35.48	61.20, 61.20, 35.27
α, β, γ (°)	90, 90, 90	90, 90, 90	90, 90, 90	90, 90, 90	90, 90, 90	90, 90, 90
Total number of observations	140194 (9706)	152869 (9459)	139496 (9520)	156378 (9812)	241587 (10881)	138579 (11039)
Unique reflections	10254 (684)	11186 (665)	10230 (675)	11438 (689)	17826 (896)	10488 (771)
Mosaicity (˚)	0	0	0	0	0	0
Multiplicity	13.7 (14.2)	13.7 (14.2)	13.6 (14.1)	13.7 (14.2)	13.6 (12.1)	13.2 (14.3)
Mean I/σ(I)	9.8 (0.3)	15.2 (0.2)	11.6 (0.4)	13.4 (0.3)	11.9 (0.3)	15.0 (1.0)
Completeness (%)	99.5 (100.0)	99.8 (96.4)	99.8 (97.5)	100.0 (99.7)	100.0 (100.0)	100.0 (100.0)
$R_{merge}{}^{b}$	0.125 (5.485)	0.082 (5.795)	0.113 (3.518)	0.088 (5.554)	0.092 (3.870)	0.127 (2.878)
$R_{meas}{}^{c}$	0.131 (5.665)	0.086 (6.163)	0.118 (3.652)	0.091 (5.761)	0.095 (1.040)	0.133 (2.985)
$R_{pim}{}^{d}$	0.036 (1.506)	0.023 (1.585)	0.045 (1.378)	0.025 (1.526)	0.026 (1.150)	0.038 (0.787)
**Refinement**						
Resolution (Å)	42.88 – 1.91	61.02 – 1.86	43.08 – 1.92	42.98 – 1.84	61.00 – 1.59	30.60 – 1.90
Reflections used	9760	10362	9804	10740	16693	10053
Free R flagged reflections	408	436	407	468	780	421
$R_{cryst}{}^{e}$	0.207	0.201	0.197	0.194	0.193	0.199
$R_{free}{}^{f}$	0.260	0.258	0.251	0.245	0.215	0.254
rmsd bonds (Å)	0.008	0.008	0.008	0.009	0.008	0.009
rmsd angles (˚)	1.539	1.524	1.594	1.591	1.542	1.628
Ramachandran plot						
Most favored (%)	100	99.12	99.12	100	99.12	100
Additionally allowed (%)	0.00	0.88	0.88	0.00	0.88	0.00
Mean B-factor (Å^2)	53.402	53.986	50.216	53.317	36.989	47.276

[a]Values for the last resolution shell are in parentheses.

[b]$R_{merge} = \Sigma_{hkl}\Sigma_i|I_i(hkl)-\langle I(hkl)\rangle| / \Sigma_{hkl}\Sigma_i I_i(hkl)$, where I(hkl) is the intensity of reflection hkl

[c]$R_{meas} = \Sigma_{hkl}(n/(n-1))^{1/2}\Sigma_i|I_i(hkl)-\langle I(hkl)\rangle| / \Sigma_{hkl}\Sigma_i I_i(hkl)$

[d]$R_{pim} = \Sigma_{hkl}(1/(n-1))^{1/2}\Sigma_i|I_i(hkl)-\langle I(hkl)\rangle| / \Sigma_{hkl}\Sigma_i I_i(hkl)$

[e]$R_{cryst} = \Sigma_{hkl} ||F_{obs}|-|F_{calc}|| / \Sigma|F_{obs}|$

[f]R_{free} is the cross-validation R-factor computed for the test set of unique reflections.

	7GAI	7GAJ	7GAK	7GAL	7GAM	7GAN
**Data collection**[a]						
Wavelength (Å)	0.92124	0.92124	0.92124	0.92124	0.92124	0.92124
Space group	P43	P43	P43	P43	P43	P43
Resolution (Å)	61.00 – 1.97	43.15 – 1.89	30.40 – 1.77	43.05 – 1.90	60.67 – 1.75	43.19 – 2.09
Last resolution shell (Å)	2.02 – 1.97	1.93 – 1.89	1.81 – 1.77	1.94 – 1.90	1.78 – 1.75	2.15 – 2.09
Unit cell parameters						
a,b,c (Å)	60.98, 60.98, 35.59	61.00, 61.00, 35.33	60.81, 60.81, 35.10	60.87, 60.87, 35.41	60.68, 60.68, 35.61	61.09, 61.09, 35.54
α, β, γ (°)	90, 90, 90	90, 90, 90	90, 90, 90	90, 90, 90	90, 90, 90	90, 90, 90
Total number of observations	128225 (9138)	145177 (9643)	173163 (9502)	141587 (9231)	180563 (9230)	106979 (8388)
Unique reflections	9444 (650)	10634 (680)	12673 (736)	10401 (656)	13277 (723)	7949 (608)
Mosaicity (˚)	0	0	0	0	0	0
Multiplicity	13.6 (14.1)	13.7 (14.2)	13.7 (12.9)	13.6 (14.1)	13.6 (12.8)	13.5 (13.8)
Mean I/σ(I)	14.7 (0.4)	10.5 (0.3)	13.6 (0.3)	9.2 (0.3)	14.3 (0.4)	10.4 (0.5)
Completeness (%)	99.9 (98.4)	100.0 (100.0)	100.0 (99.4)	99.9 (99.2)	99.9 (98.3)	99.9 (99.3)
R_{merge}[b]	0.076 (2.872)	0.113 (5.099)	0.104 (6.109)	0.118 (3.840)	0.068 (2.668)	0.124 (3.388)
R_{meas}[c]	0.079 (2.980)	0.118 (5.288)	0.109 (6.361)	0.123 (3.985)	0.070 (2.878)	0.129 (3.517)
R_{pim}[d]	0.022 (0.793)	0.032 (1.395)	0.030 (1.764)	0.033 (1.059)	0.026 (1.122)	0.035 (0.937)
**Refinement**						
Resolution (Å)	61.00 – 1.97	43.15 – 1.89	30.40 – 1.77	43.05 – 1.90	60.67 – 1.75	43.19 – 2.09
Reflections used	9017	9956	12022	9695	12594	7594
Free R flagged reflections	361	419	549	402	583	299
R_{cryst}[e]	0.207	0.184	0.200	0.193	0.197	0.189
R_{free}[f]	0.281	0.229	0.250	0.251	0.234	0.256
rmsd bonds (Å)	0.007	0.008	0.009	0.006	0.010	0.007
rmsd angles (˚)	1.541	1.550	1.619	1.424	1.626	1.482
Ramachandran plot						
Most favored (%)	99.12	98.23	100	100	100	98.23
Additionally allowed (%)	0.88	1.77	0.00	0.00	0.00	1.77
Mean B-factor (Å^2)	62.225	50.720	49.773	52.913	48.775	59.148

[a]Values for the last resolution shell are in parentheses.

[b]$R_{merge} = \Sigma_{hkl}\Sigma_i|I_i(hkl)-\langle I(hkl)\rangle| / \Sigma_{hkl}\Sigma_i I_i(hkl)$, where I(hkl) is the intensity of reflection hkl

[c]$R_{meas} = \Sigma_{hkl}(n/(n-1))^{1/2}\Sigma_i|I_i(hkl)-\langle I(hkl)\rangle| / \Sigma_{hkl}\Sigma_i I_i(hkl)$

[d]$R_{pim} = \Sigma_{hkl}(1/(n-1))^{1/2}\Sigma_i|I_i(hkl)-\langle I(hkl)\rangle| / \Sigma_{hkl}\Sigma_i I_i(hkl)$

[e]$R_{cryst} = \Sigma_{hkl} ||F_{obs}|-|F_{calc}|| / \Sigma|F_{obs}|$

[f]R_{free} is the cross-validation R-factor computed for the test set of unique reflections.

	7GAO	7GAP	7GAQ	7GAR	7GAS
**Data collection**[a]					
Wavelength (Å)	0.92124	0.92124	0.92124	0.92124	0.92124
Space group	P43	P43	P43	P43	P43
Resolution (Å)	30.56 – 1.69	30.63 – 1.68	30.69 – 2.14	30.58 – 2.07	30.66 – 1.91
Last resolution shell (Å)	1.72 – 1.69	1.71 – 1.68	2.20 – 2.14	2.13 – 2.07	1.96 – 1.91
Unit cell parameters					
a,b,c (Å)	61.07, 61.07, 35.30	61.21, 61.21, 35.38	61.18, 61.18, 35.47	61.03, 61.03, 35.29	60.85, 60.85, 35.50
α, β, γ (°)	90, 90, 90	90, 90, 90	90, 90, 90	90, 90, 90	90, 90, 90
Total number of observations	201357 (10272)	207746 (10646)	98511 (7335)	109090 (8464)	140622 (9796)
Unique reflections	14738 (749)	15116 (760)	7415 (532)	8083 (600)	10191 (681)
Mosaicity (˚)	0	0	0	0	0
Multiplicity	13.7 (13.7)	13.7 (14.0)	13.3 (13.8)	13.5 (14.1)	13.8 (14.4)
Mean I/σ(I)	10.2 (0.5)	9.9 (0.3)	6.5 (0.7)	8.6 (0.3)	10.8 (0.3)
Completeness (%)	99.9 (99.1)	99.6 (98.8)	100.0 (100.0)	99.8 (98.8)	99.2 (98.2)
R_{merge}[b]	0.262 (13.352)	0.153 (5.552)	0.377 (4.262)	0.173 (7.250)	0.141 (7.310)
R_{meas}[c]	0.273 (13.865)	0.159 (5.761)	0.392 (4.426)	0.181 (7.521)	0.146 (7.580)
R_{pim}[d]	0.074 (3.716)	0.043 (1.530)	0.107 (1.187)	0.050 (1.991)	0.040 (1.989)
**Refinement**					
Resolution (Å)	30.56 – 1.69	30.63 – 1.68	30.69 – 2.14	30.57 – 2.07	30.68 – 1.91
Reflections used	14048	14358	7114	7677	9624
Free R flagged reflections	660	674	289	304	400
R_{cryst}[e]	0.192	0.203	0.188	0.191	0.219
R_{free}[f]	0.218	0.244	0.244	0.264	0.276
rmsd bonds (Å)	0.007	0.008	0.008	0.007	0.008
rmsd angles (˚)	1.459	1.494	1.509	1.507	1.549
Ramachandran plot					
Most favored (%)	100	99.12	100	97.35	99.12
Additionally allowed (%)	0.00	0.88	0.00	1.77	0.88
Mean B-factor (Å^2)	35.080	35.028	43.990	62.764	54.217

[a]Values for the last resolution shell are in parentheses.

[b]$R_{merge} = \Sigma_{hkl}\Sigma_i|I_i(hkl)-\langle I(hkl)\rangle| / \Sigma_{hkl}\Sigma_i I_i(hkl)$, where I(hkl) is the intensity of reflection hkl

[c]$R_{meas} = \Sigma_{hkl}(n/(n-1))^{1/2}\Sigma_i|I_i(hkl)-\langle I(hkl)\rangle| / \Sigma_{hkl}\Sigma_i I_i(hkl)$

[d]$R_{pim} = \Sigma_{hkl}(1/(n-1))^{1/2}\Sigma_i|I_i(hkl)-\langle I(hkl)\rangle| / \Sigma_{hkl}\Sigma_i I_i(hkl)$

[e]$R_{cryst} = \Sigma_{hkl} ||F_{obs}|-|F_{calc}|| / \Sigma|_{Fobs}|$

[f]R_{free} is the cross-validation R-factor computed for the test set of unique reflections.

Supplementary Table 5: **Small molecule screening data**

Category	Parameter	Description
Assay	Type of assay	Crystallographic Fragment Screening
	Target	Microtubule-associated proteins 1A/1B light chain 3B (Uniprot ID: Q9GZQ8)
	Primary measurement	Detection of bound fragments and possibly identify novel binding sites
	Key reagents	• DSi Poised fragment library solubilized in 100% DMSO<br>• MAP1LC3B protein in 30 mM HEPES pH 7.5, 100 mM NaCl, 0.5 mM TCEP, 5% Glycerol<br>• Crystallization condition: 36% PEG 8000, 0.1M acetate pH 4.7
	Assay protocol	• https://dx.doi.org/10.3791/62414<br>• Also detailed in out manuscript under subheading "Fragment Screening and Structure Solution" in "Methods" section
	Additional comments	Some crystals are bound to re-solubilize while soaking. They can be repeated with lower DMSO concentration and/or soaking durations.
Library	Library size	988 fragment molecules
	Library composition	Poised fragments contain at least one functional group which can be synthesised using a robust, well-characterised reaction. Reactions include amide couplings, Suzuki-type aryl-aryl couplings and reductive aminations.
	Source	Xchem (Diamond Light Source, UK); Also available from Enamine
	Additional comments	https://www.diamond.ac.uk/Instruments/Mx/Fragment-Screening/Fragment-Libraries/DSi-Poised-Library.html
Screen	Format	• Fragment source plate: 1536 LDV ECHO-compatible plate<br>• Crystallization plates: 96 well 3-drop SWISSCI plates
	Concentration(s) tested	• 100 mM (20% DMSO)<br>• 50.6 mM (10% DMSO)
	Plate controls	Crystal stability over time and DMSO concentrations was tested with DMSO soak only
	Reagent/ compound dispensing system	ECHO Liquid Handler
	Detection instrument and software	x-ray detector: DECTRIS PILATUS 6M
	Assay validation/QC	Structures solved through XChemExplorer (XCE) pipeline
	Correction factors	Data scaling (Corrections due to beam fluctuations, crystal decay, absorption)
	Normalization	n.a.
	Additional comments	Information on Xchem facility can be accessed here:<br>https://www.diamond.ac.uk/Instruments/Mx/Fragment-Screening/The-XChem-Pipeline.html
Post-HTS analysis	Hit criteria	Observable density in event maps
	Hit rate	21 this were obtained out of 988
	Additional assay(s)	n.a.
	Confirmation of hit purity and structure	QC information can be downloaded from the link: https://www.diamond.ac.uk/Instruments/Mx/Fragment-Screening/Fragment-Libraries/DSi-Poised-Library.html
	Additional comments	n.a.

Supplementary References:

1 Li, Z. *et al.* Allele-selective lowering of mutant HTT protein by HTT-LC3 linker compounds. *Nature* **575**, 203-209 (2019). https://doi.org:10.1038/s41586-019-1722-1

2 Fan, S. *et al.* Inhibition of Autophagy by a Small Molecule through Covalent Modification of the LC3 Protein. *Angew Chem Int Ed Engl* **60**, 26105-26114 (2021). https://doi.org:10.1002/anie.202109464

3 Tsuganezawa, K. *et al.* Two-colored fluorescence correlation spectroscopy screening for LC3-P62 interaction inhibitors. *J Biomol Screen* **18**, 1103-1109 (2013). https://doi.org:10.1177/1087057113492200

4 Hartmann, M. *et al.* Demonstrating Ligandability of the LC3A and LC3B Adapter Interface. *J Med Chem* **64**, 3720-3746 (2021). https://doi.org:10.1021/acs.jmedchem.0c01564

5 Wirth, M. *et al.* Molecular determinants regulating selective binding of autophagy adapters and receptors to ATG8 proteins. *Nat Commun* **10**, 2055 (2019). https://doi.org:10.1038/s41467-019-10059-6

6 Rogov, V. V. *et al.* Phosphorylation of the mitochondrial autophagy receptor Nix enhances its interaction with LC3 proteins. *Sci Rep* **7**, 1131 (2017). https://doi.org:10.1038/s41598-017-01258-6

7 Stolz, A. *et al.* Fluorescence-based ATG8 sensors monitor localization and function of LC3/GABARAP proteins. *EMBO J* **36**, 549-564 (2017). https://doi.org:10.15252/embj.201695063

8 Holdgaard, S. G. *et al.* Selective autophagy maintains centrosome integrity and accurate mitosis by turnover of centriolar satellites. *Nat Commun* **10**, 4176 (2019). https://doi.org:10.1038/s41467-019-12094-9

9 Di Rita, A. *et al.* HUWE1 E3 ligase promotes PINK1/PARKIN-independent mitophagy by regulating AMBRA1 activation via IKKalpha. *Nat Commun* **9**, 3755 (2018). https://doi.org:10.1038/s41467-018-05722-3

10 Dong, G. *et al.* Ispinesib as an Effective Warhead for the Design of Autophagosome-Tethering Chimeras: Discovery of Potent Degraders of Nicotinamide Phosphoribosyltransferase (NAMPT). *J Med Chem* **65**, 7619-7628 (2022). https://doi.org:10.1021/acs.jmedchem.1c02001

11 Hanke, T. *et al.* Identification of pirinixic acid derivatives bearing a 2-aminothiazole moiety combines dual PPARalpha/gamma activation and dual 5-LO/mPGES-1 inhibition. *Bioorg Med Chem Lett* **24**, 3757-3763 (2014). https://doi.org:10.1016/j.bmcl.2014.06.077

12 Hanke, T. *et al.* Synthesis and pharmacological characterization of benzenesulfonamides as dual species inhibitors of human and murine mPGES-1. *Bioorg Med Chem* **21**, 7874-7883 (2013). https://doi.org:10.1016/j.bmc.2013.10.006

13 Hanke, T. *et al.* Development and Characterization of Type I, Type II, and Type III LIM-Kinase Chemical Probes. *J Med Chem* **65**, 13264-13287 (2022). https://doi.org:10.1021/acs.jmedchem.2c01106

14 Hanke, T. *et al.* A Selective Modulator of Peroxisome Proliferator-Activated Receptor gamma with an Unprecedented Binding Mode. *J Med Chem* **63**, 4555-4561 (2020). https://doi.org:10.1021/acs.jmedchem.9b01786

15 Hanke, T. *et al.* Aminothiazole-featured pirinixic acid derivatives as dual 5-lipoxygenase and microsomal prostaglandin E2 synthase-1 inhibitors with improved potency and efficiency in vivo. *J Med Chem* **56**, 9031-9044 (2013). https://doi.org:10.1021/jm401557w
