# Supplementary material for: Critical assessment of LC3/GABARAP ligands used for degrader development and ligandability of LC3/GABARAP binding pockets
Source: Nat Commun. 2024 Nov 25;15:10204. doi: 10.1038/s41467-024-54409-5 (PMC11589570; doi:10.1038/s41467-024-54409-5)
Supplement: Supplementary file 2 — Reporting Summary [file 41467_2024_54409_MOESM2_ESM.pdf]

nature portfolio

Corresponding author(s): Stefan Knapp, Vladimir V. Rogov

Last updated by author(s): Sep 17, 2024

Reporting Summary

Nature Portfolio wishes to improve the reproducibility of the work that we publish. This form provides structure for consistency and transparency in reporting. For further information on Nature Portfolio policies, see our Editorial Policies and the Editorial Policy Checklist.

Statistics

For all statistical analyses, confirm that the following items are present in the figure legend, table legend, main text, or Methods section.

n/a	Confirmed	
☐	☒	The exact sample size (*n*) for each experimental group/condition, given as a discrete number and unit of measurement
☐	☒	A statement on whether measurements were taken from distinct samples or whether the same sample was measured repeatedly
☒	☐	The statistical test(s) used AND whether they are one- or two-sided<br>*Only common tests should be described solely by name; describe more complex techniques in the Methods section.*
☒	☐	A description of all covariates tested
☒	☐	A description of any assumptions or corrections, such as tests of normality and adjustment for multiple comparisons
☐	☒	A full description of the statistical parameters including central tendency (e.g. means) or other basic estimates (e.g. regression coefficient) AND variation (e.g. standard deviation) or associated estimates of uncertainty (e.g. confidence intervals)
☒	☐	For null hypothesis testing, the test statistic (e.g. *F*, *t*, *r*) with confidence intervals, effect sizes, degrees of freedom and *P* value noted<br>*Give P values as exact values whenever suitable.*
☒	☐	For Bayesian analysis, information on the choice of priors and Markov chain Monte Carlo settings
☒	☐	For hierarchical and complex designs, identification of the appropriate level for tests and full reporting of outcomes
☒	☐	Estimates of effect sizes (e.g. Cohen's *d*, Pearson's *r*), indicating how they were calculated

Our web collection on statistics for biologists contains articles on many of the points above.

Software and code

Policy information about availability of computer code

Data collection	FPA: No software was used for data collection<br>ITC: NanoITC instrument package (TA Instruments, New Castle, USA)<br>DSF: QuantStudio5 package (Applied Biosystems)<br>Spectral shift: Dianthus package (NanoTemper Technologies GmbH, Germany)<br>Mass spectroscopy: a) LC/MS - Agilent 6230 TOF LC/MS package, b) LC-MS/MS - Thermo Scientific Xcalibur<br>Nano BRET: No software was used for data collection<br>Cell growth assay: IncuCyte S3 package (Sartiorius, Germany)<br>NMR: TopSpin v3.6.2, (Bruker Biospin, Germany)
Data analysis	FPA: GraphPad Prism 9.3 (graphic representation), Excel (fitting)<br>ITC: TA Instruments NanoAnalyze package, GraphPad Prism 9.3 (graphic representation)<br>DSF Protein Thermal Shift software (Applied Biosystems)<br>Spectral shift: DI.Screening Analysis Software (v.2.0.4) (NanoTemper Technologies GmbH, Germany)<br>Mass spectroscopy: a) LC/MS - BioConfirm B.08.00, b) MaxQuant (v 1.6.1.0)<br>Nano BRET: GraphPad Prism 9.3 (graphic representation), Excel (fitting)<br>Cell growth assay: IncuCyte S3 package (Sartiorius, Germany), Excel for graphic representation)<br>Virtual screening: SeeSAR (BioSolveIT), AutoDock-GPU, Autodock<br>Similarity search: infiniSee suite (BioSolveIT)<br>NMR: Sparky v3.114 (graphic representation), Excel (fitting), PyMOL 0.99rc6 (structural analysis)

X-ray: XChem Explorer pipeline software, PanDDA, REFMAC

For manuscripts utilizing custom algorithms or software that are central to the research but not yet described in published literature, software must be made available to editors and reviewers. We strongly encourage code deposition in a community repository (e.g. GitHub). See the Nature Portfolio guidelines for submitting code & software for further information.

Data

Policy information about availability of data

All manuscripts must include a data availability statement. This statement should provide the following information, where applicable:

- Accession codes, unique identifiers, or web links for publicly available datasets
- A description of any restrictions on data availability
- For clinical datasets or third party data, please ensure that the statement adheres to our policy

Atomic coordinates and structure factors were deposited in the Protein Data Bank with accession codes: 8Q53 for LC3B apo-form, 7GAU for LC3B "ground state", and 7GA8-7GA9, 7GAA- 7GAS for LC3B complexes with corresponding ligands.
All data needed to evaluate the conclusions of this study are presented in the paper and Supplementary information files. Requests for materials should be addressed to knapp@pharmchem.uni-frankfurt.de and rogov@pharmchem.uni-frankfurt.de

Research involving human participants, their data, or biological material

Policy information about studies with human participants or human data. See also policy information about sex, gender (identity/presentation), and sexual orientation and race, ethnicity and racism.

Reporting on sex and gender	No research involving human participants has been conducted
Reporting on race, ethnicity, or other socially relevant groupings	No research involving human participants has been conducted
Population characteristics	No research involving human participants has been conducted
Recruitment	No research involving human participants has been conducted
Ethics oversight	No research involving human participants has been conducted

Note that full information on the approval of the study protocol must also be provided in the manuscript.

Field-specific reporting

Please select the one below that is the best fit for your research. If you are not sure, read the appropriate sections before making your selection.

☒ Life sciences ☐ Behavioural & social sciences ☐ Ecological, evolutionary & environmental sciences

For a reference copy of the document with all sections, see nature.com/documents/nr-reporting-summary-flat.pdf

Life sciences study design

All studies must disclose on these points even when the disclosure is negative.

Sample size	Based on standard practice, 3 replicates were used the screenings and biophysical experiments. 2 technical replicates were used for spectral shift assay.
Data exclusions	No data were excluded.
Replication	All experiments were conducted 2-3 times, beside ITC and NMR experiments. All attempts at replication were successful.
Randomization	For most experiments, the samples were not randomly assigned. There was no need for group allocation or randomization; all samples were independently measured in controlled manner.
Blinding	Since individual experiments were set up and performed by specific researches, it was impractical to assign other personnel to implement blinding in the large and time-sensitive experimental setups. However, whenever possible, additional one or two researches confirmed the results.

Reporting for specific materials, systems and methods

We require information from authors about some types of materials, experimental systems and methods used in many studies. Here, indicate whether each material, system or method listed is relevant to your study. If you are not sure if a list item applies to your research, read the appropriate section before selecting a response.

Materials & experimental systems

n/a	Involved in the study
☒	☐ Antibodies
☐	☒ Eukaryotic cell lines
☒	☐ Palaeontology and archaeology
☒	☐ Animals and other organisms
☒	☐ Clinical data
☒	☐ Dual use research of concern
☒	☐ Plants

Methods

n/a	Involved in the study
☒	☐ ChIP-seq
☒	☐ Flow cytometry
☒	☐ MRI-based neuroimaging

Eukaryotic cell lines

Policy information about cell lines and Sex and Gender in Research

Cell line source(s)	All cell lines obtained from ATCC, no engeneered cell lines used. Common RPE1 (hTERT RPE-1, ATCC CLR_4000) and U2OS (ATCC HTB-96) cell lines were used in this study to generate grow curves. HEK293T cells (ATCC CRL-11268) were used in proteomics and nanoBRET experiments.
Authentication	Cell line were not authenticated after delivery.
Mycoplasma contamination	All cell lines were regulary tested for Mycoplasma contamination during routine screening procedures, testings were negative.
Commonly misidentified lines (See ICLAC register)	No commonly misidentified cell lines were used in the study.

Plants

Seed stocks	*Report on the source of all seed stocks or other plant material used. If applicable, state the seed stock centre and catalogue number. If plant specimens were collected from the field, describe the collection location, date and sampling procedures.*
Novel plant genotypes	*Describe the methods by which all novel plant genotypes were produced. This includes those generated by transgenic approaches, gene editing, chemical/radiation-based mutagenesis and hybridization. For transgenic lines, describe the transformation method, the number of independent lines analyzed and the generation upon which experiments were performed. For gene-edited lines, describe the editor used, the endogenous sequence targeted for editing, the targeting guide RNA sequence (if applicable) and how the editor was applied.*
Authentication	*Describe any authentication procedures for each seed stock used or novel genotype generated. Describe any experiments used to assess the effect of a mutation and, where applicable, how potential secondary effects (e.g. second site T-DNA insertions, mosiacism, off-target gene editing) were examined.*
